# C≡N and N≡O Bond Cleavages of Acetonitrile and Nitrosyl Ligands at a Dimolybdenum Center to Render Ethylidyne and Acetamidinate Ligands

**DOI:** 10.1021/acs.inorgchem.3c03697

**Published:** 2024-02-02

**Authors:** M. Angeles Alvarez, M. Esther García, Daniel García-Vivó, Ana M. Guerra, Miguel A. Ruiz

**Affiliations:** Departamento de Química Orgánica e Inorgánica, Instituto Universitario de Química Organometálica “Enrique Moles”, Universidad de Oviedo, E33071 Oviedo, Spain

## Abstract

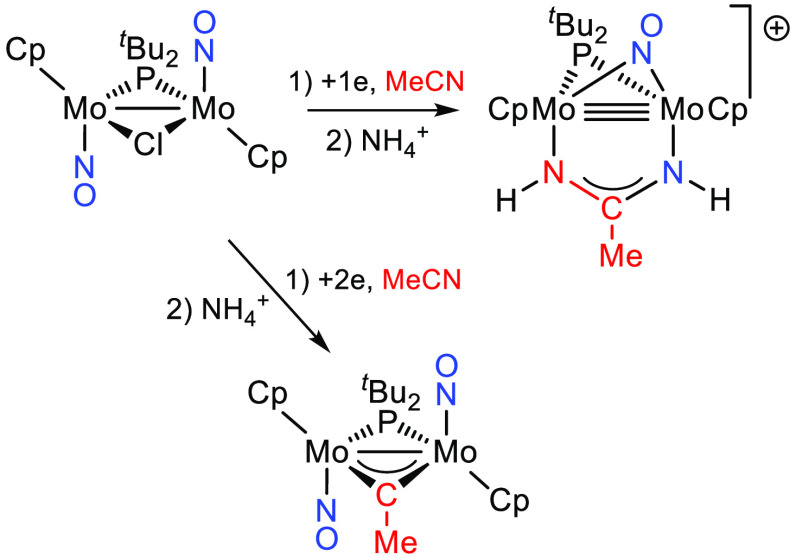

Extended reduction of [Mo_2_Cp_2_(μ-Cl)(μ-P^*t*^Bu_2_)(NO)_2_] (**1**) with Na(Hg) in acetonitrile (MeCN) at room temperature resulted in an unprecedented full cleavage of the C≡N bond of a coordinated MeCN molecule to yield the vinylidene derivative Na[Mo_2_Cp_2_(μ-P^*t*^Bu_2_)(μ-CCH_2_)(NO)_2_], which upon protonation with (NH_4_)PF_6_ gave the ethylidyne complex [Mo_2_Cp_2_(μ-P^*t*^Bu_2_)(μ-CMe)(NO)_2_] [Mo1–Mo2 = 2.9218(2) Å] in a selective and reversible way. Controlled reduction of **1** at 273 K yielded instead, after protonation, the 30-electron acetamidinate complex [Mo_2_Cp_2_(μ-P^*t*^Bu_2_)(μ-κ*N*:κ*N*′-HNCMeNH)(μ-NO)]PF_6_ [Mo1–Mo2 = 2.603(2) Å], in a process thought to stem from the paramagnetic MeCN-bridged intermediate [Mo_2_Cp_2_(μ-P^*t*^Bu_2_)(μ-NCMe)(NO)_2_], followed by a complex sequence of elementary steps including cleavage of the N≡O bond of a nitrosyl ligand.

## Abstract 2

Two-electron reduction of the chloride-bridged complex [Mo_2_Cp_2_(μ-Cl)(μ-P^*t*^Bu_2_)(NO)_2_] in acetonitrile (MeCN) at room temperature resulted in an unprecedented full cleavage of the C≡N bond of a coordinated MeCN molecule rendering a vinylidene-bridged intermediate, yielding, after protonation, the ethylidyne complex [Mo_2_Cp_2_(μ-P^*t*^Bu_2_)(μ-CMe)(NO)_2_]. One-electron reduction at 273 K yielded instead, after protonation, the acetamidinate-bridged cation [Mo_2_Cp_2_(μ-P^*t*^Bu_2_)(μ-κ*N*:κ*N*′-HNCMeNH)(μ-NO)]^+^, in a multistep process likely involving cleavage of the strong N≡O bond of a nitrosyl ligand and nitrene/iminoacyl coupling.

There have been for some time investigations on the chemistry of binuclear transition-metal complexes bearing different types of unsaturation (coordinative, electronic, or both) on the hypothesis that these are molecules able to induce activation and cleavage of the strong N≡O bond of nitric oxide at a dimetal site.^1−4^ These are processes of academic interest in the context of the rich chemistry of nitrosyl complexes^5^ and also because nitric oxide is an important air pollutant requiring catalytic, metal-mediated abatement, a process that involves degradation of the nitric oxide molecule while interacting with one or more metal atoms.^6^

Recently, we reported the synthesis of the Na^+^ salt of the unsaturated anion [W_2_Cp_2_(μ-PPh_2_)(NO)_2_]^−^ upon reduction of [W_2_Cp_2_(μ-I)(μ-PPh_2_)(NO)_2_] with Na(Hg) in acetonitrile (MeCN).^7^ This 32-electron complex, as well as the corresponding hydride derivative [W_2_Cp_2_(μ-H)(μ-PPh_2_)(NO)_2_], was a highly reactive species, allowing the synthesis of a large diversity of derivatives,^7,8^ but no N–O bond cleavage processes were observed in any of the corresponding reactions, so we turned to inspect the chemistry of related dimolybdenum complexes. First we found that reduction reactions of [Mo_2_Cp_2_(μ-Cl)(μ-PPh_2_)(NO)_2_] were of poor selectivity and failed to yield the desired unsaturated species. Then we decided to investigate the reduction reactions of the analogous P^*t*^Bu_2_ complex [Mo_2_Cp_2_(μ-Cl)(μ-P^*t*^Bu_2_)(NO)_2_] (**1**) with the expectation that the bulky ^*t*^Bu groups might provide additional steric protection (hence, enhanced stability) to the targeted unsaturated anion [Mo_2_Cp_2_(μ-P^*t*^Bu_2_)(NO)_2_]^−^ and the corresponding hydride derivative. As shown below, these reactions failed to yield the soughtafter complexes but instead unveiled the operation of unexpected processes taking place under mild conditions, including cleavage of the N≡O bond of a nitrosyl ligand and N–C coupling to eventually yield an acetamidinate ligand, and cleavage of the C≡N bond of a MeCN ligand to give vinylidene and then ethylidyne ligands. While all of these processes are themselves unusual, we note that previous examples of cleavage of the C≡N bond of nitriles by reactive metal complexes mostly led to nitride and/or carbyne derivatives.^9^ However, examples of the generation of a vinylidene group from MeCN are restricted, to our knowledge, to the recently reported reaction of laser-ablated B atoms with MeCN on a solid neon matrix under full arc irradiation.^10^

Compound **1** was prepared following the method previously developed for the ditungsten analogue [W_2_Cp_2_(μ-I)(μ-PPh_2_)(NO)_2_].^7^ To this purpose, the known dicarbonyl complex [Mo_2_Cp_2_(μ-Cl)(μ-P^*t*^Bu_2_)(CO)_2_]^11^ was first reacted with NO (5% in Ar, 1 atm) in a tetrahydrofuran (THF) solution at 233 K to give the monocarbonyl intermediate [Mo_2_Cp_2_Cl(μ-P^*t*^Bu_2_)(CO)(NO)_2_] (not isolated),^12^ which was then refluxed in toluene to give **1** in 63% yield (Scheme 1). Spectroscopic data for this product (see the Supporting Information, SI) were comparable to those of the mentioned ditungsten complex, except for the expected changes associated with the replacements W/Mo and Ph/^*t*^Bu.

**Scheme 1:**
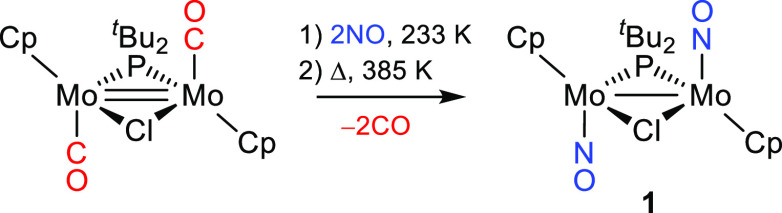
Synthesis of Compound **1**

Reduction reactions of **1** were particularly sensitive to experimental conditions such as the solvent, reducing reagent, temperature, and reaction time. Reactions with Na(Hg) in THF were of poor selectivity and were not further explored. In contrast, analogous reactions in MeCN proved to be more selective, although they were far from yielding the targeted anion [Mo_2_Cp_2_(μ-P^*t*^Bu_2_)(NO)_2_]^−^. Actually, reaction of **1** with Na(Hg) in MeCN at room temperature for 40 min yielded the Na^+^ salt of the anionic vinylidene complex [Mo_2_Cp_2_(μ-P^*t*^Bu_2_)(μ-CCH_2_)(NO)_2_]^−^ (**2-Na**) as a major product, which upon reaction with (NH_4_)PF_6_ yielded the ethylidyne derivative [Mo_2_Cp_2_(μ-P^*t*^Bu_2_)(μ-CMe)(NO)_2_] (**3**) selectively (Scheme 2). The latter process could be reversed upon reaction of **3** with a strong base such as 1,8-diazabicyclo[5.4.0]undec-7-ene (DBU), which yielded the salt (DBUH)[Mo_2_Cp_2_(μ-P^*t*^Bu_2_)(μ-CCH_2_)(NO)_2_] (**2-DBUH**) selectively.

**Scheme 2:**
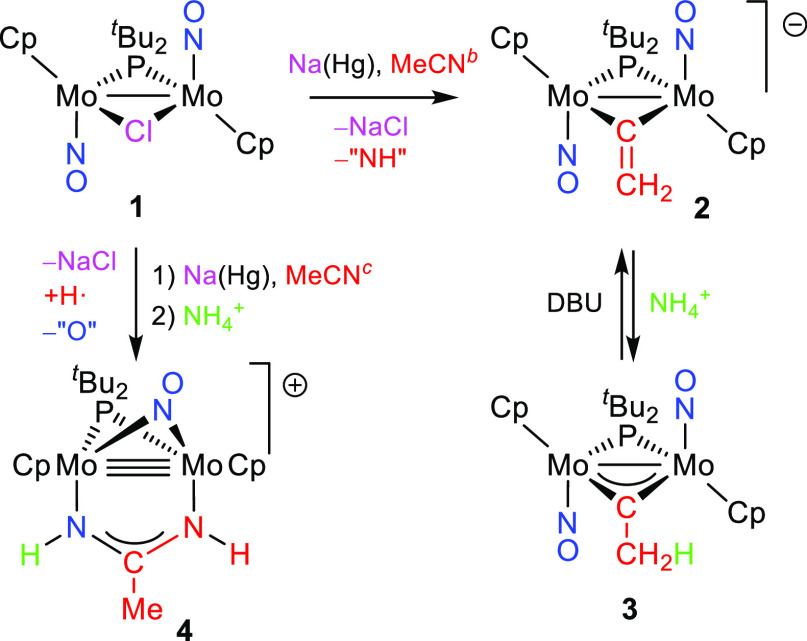
Reduction of Compound **1** in MeCN Counterions are Na^+^ or (DBUH)^+^ for **2**, PF_6_^–^ for **4**, and NH_4_^+^. Reduction at room temperature for 40 min. Reduction at 273 K for 15 min.

The structure of **3** (Figure 1) is comparable to that of the benzylidyne dicarbonyl complex [Mo_2_Cp_2_(μ-PCy_2_)(μ-CPh)(CO)_2_],^13^ although its electron-precise nature accounts for its much longer intermetallic distance [2.9218(2) vs 2.666(1) Å], while higher Mo–P lengths and puckering of the central MPMC ring in **3** (P–Mo–Mo–C = 148.5°) can be attributed to the larger steric demands of the P^*t*^Bu_2_ bridge.^11^ The deep-purple color of **3** is unexpected for a 34-electron complex. According to a time-dependent density functional theory (DFT) calculation, this color would originate in a main visible absorption at ca. 510 nm (expt 540 nm in a CH_2_Cl_2_ solution) due to transits to the lowest unoccupied molecular orbital (LUMO) from the highest occupied molecular orbital (HOMO) and other closely placed frontier molecular orbitals (Figures S18–S20). The low HOMO–LUMO gap of 2.08 eV, in turn, might be related to the strong deshielding of the bridging carbyne carbon in **3**,^14^ which gives rise to a ^13^C NMR resonance at 487.8 ppm, a chemical shift among the highest reported for diamagnetic complexes [cf. 490.2 ppm for [Fe_2_Cp_2_(μ-CH)(μ-CO)(CO)_2_](PF_6_)].^15^

**Figure 1:**
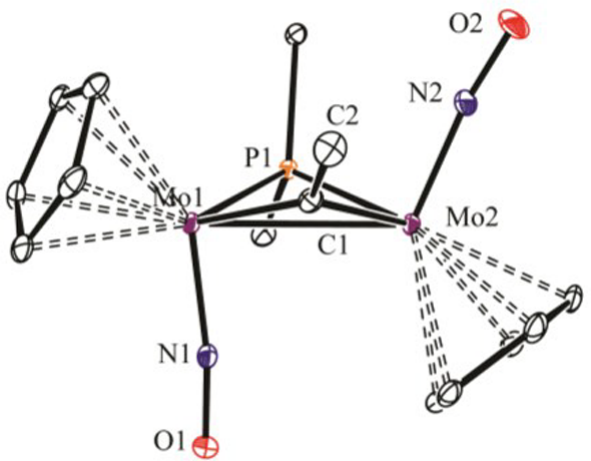
ORTEP diagram (30% probability) of compound **3**, with ^*t*^Bu groups (except their C^1^ atoms) and H atoms omitted. Selected bond lengths (Å): Mo1–Mo2 = 2.9218(2); Mo1–P1 = 2.4837(4); Mo1–C1 = 1.997(2); Mo1–N1 = 1.797(2).

The presence in **2** of a vinylidene ligand bridging the dimetal center through the carbenic C atom is indicated by a strongly deshielded resonance at 275.5 ppm in its ^13^C NMR spectrum and by NMR resonances in the aromatic region indicative of uncoordinated CH_2_ groups (δ_C_ = 124.2 ppm; δ_H_ = 6.88 ppm), all of them comparable to the corresponding resonances in the related ruthenium complexes [Ru_2_Cp_2_(μ-CCH_2_)(μ-CO)(CO)_2_]^16^ and [Ru_2_Cp*_2_(μ-CCH_2_)(μ-NPh)].^17^ We note that the latter complexes were also protonated at their CH_2_ groups to give the corresponding ethylidyne derivatives. The proposed structure for **2** is also in agreement with DFT calculations for this anion (see the SI), which rendered an optimized structure with the CCH_2_ ligand symmetrically bridging the metal atoms through its carbenic carbon (Mo–C ca. 2.10 Å).

To ascertain the origin of the bridging C_2_ ligands present in complexes **2** and **3**, we carried out the reduction reaction of **1** using MeCN-*d*_3_ as the solvent, to find that the final carbyne ligand had the expected deuteration degree (**3**-*d*_2_). In contrast, no deuteration was observed when the reduction and protonation steps were performed in MeCN and MeCN-*d*_3_, respectively. All of this proves that the C_2_ ligands in **2** and **3** have their origin in the solvent, which has been denitrogenated along the reduction reaction. It is likely that a MeCN molecule binds the dimetal center following the release of chloride caused by the first electron transfer to give a paramagnetic intermediate [Mo_2_Cp_2_(μ-P^*t*^Bu_2_)(μ-NCMe)(NO)_2_] (**A**) undetectable by NMR spectroscopy. Further reduction of this radical should give a detectable diamagnetic MeCN complex [Mo_2_Cp_2_(μ-P^*t*^Bu_2_)(μ-NCMe)(NO)_2_]^−^ (**B**), but the latter seems to undergo somehow a fast release of N and H atoms to give the vinylidene ligand found in **2** because no intermediates are detected when monitoring the formation of **2** by ^31^P NMR spectroscopy. According to DFT calculations, the most likely structures for intermediates **A** and **B** would bear *bridging* rather than *terminal* MeCN ligands (Scheme 3). In the case of radical **A**, two almost isoergonic isomers **A1** and **A2** were found, with coordination modes that we might describe as μ-κN:κN and μ-κC:η^2^, respectively (see the SI). In the case of anion **B**, the μ-κC:κN coordination mode is preferred over the other alternatives. All of these are coordination modes not identified structurally for nitrile ligands so far. We also note that the spin densities in isomers **A1** and **A2** are mainly located respectively at the C and N atoms of the MeCN ligand (see the SI), a circumstance favoring atom-abstraction reactions at any of these two sites (see later). Interestingly, the strong C≡N triple bond of MeCN is significantly weakened as a result of the bridging coordination in all of these intermediate species, with computed C–N distances of 1.225 Å (**A1**), 1.289 Å (**A2**), and 1.275 Å (**B**), the latter two being slightly above the reference figure of ca. 1.26 Å for C=N double bonds^18^ and well above the value computed for free MeCN (1.165 Å). Further studies are now in progress to check whether these bridging coordination modes are relevant to facilitate the H-shift that would prepare the MeCN molecule to undergo the C–N bond cleavage that eventually renders the vinylidene group. It is interesting to note that the C≡N bond cleavage observed in the reaction of B atoms with MeCN also involves a H-shift, eventually yielding linear HBNBCCH_2_ molecules.^10^

**Scheme 3:**
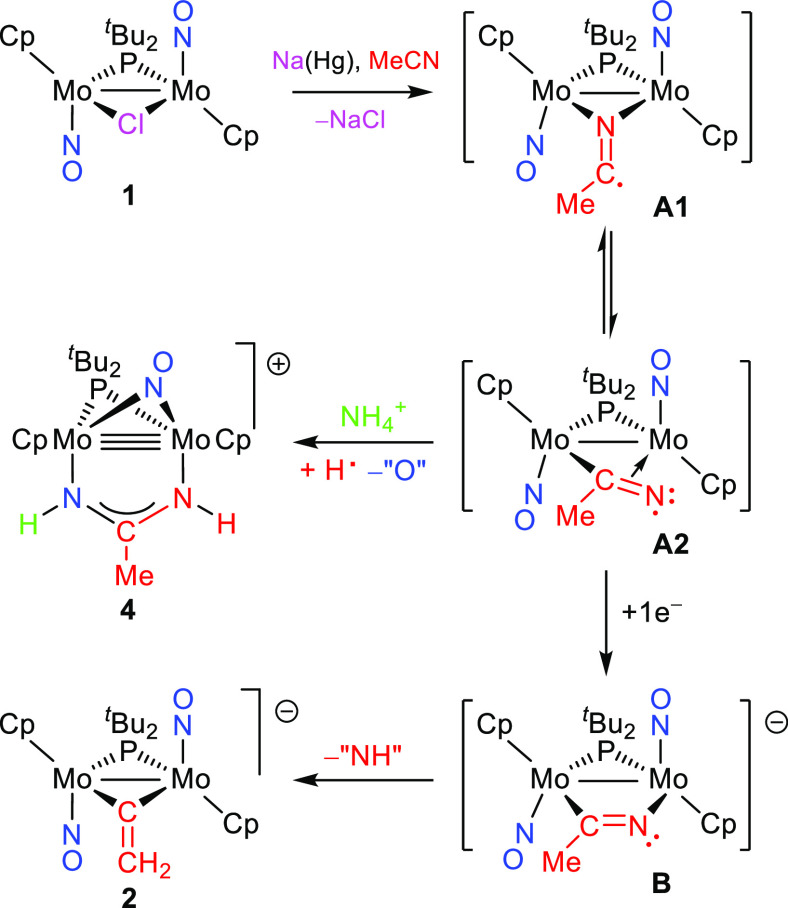
Proposed Intermediates in the Reduction of **1**

The reduction of **1** gave a very different output when it was performed at 273 K for a shorter reaction time of about 15 min before the protonation step with (NH_4_)PF_6_. At this stage, the IR spectrum of the solution displays a new strong N–O stretch at 1567 cm^–1^, only 8 cm^–1^ below that of **1**, which is consistent with the presence of a neutral species such as radical **A** (cf. 1566 cm^–1^ for **3**). Under these conditions, the major product formed after protonation was the PF_6_^–^ salt of the acetamidinate complex [Mo_2_Cp_2_(μ-P^*t*^Bu_2_)(μ-κ*N*:κ*N*′-HNCMeNH)(μ-NO)]^+^ (**4-PF**_**6**_), along with small amounts of **3** and other unidentified species (Scheme 2). When performed in MeCN-*d*_3_, the reaction described above yielded [Mo_2_Cp_2_(μ-P^*t*^Bu_2_)(μ-κ*N*:κ*N*′-HNCCD_3_ND)(μ-NO)]PF_6_ (**4-PF**_**6**_-*d*_4_), indicating that one of the NH H atoms stems from the solvent. When performed in 98% ^15^N-enriched acetonitrile (MeCN*), the above reaction yielded [Mo_2_Cp_2_(μ-P^*t*^Bu_2_)(μ-κ*N*:κ*N*′-HNCMeN*H)(μ-NO)]PF_6_, as determined by ^1^H NMR (Figure S28), indicating that only one of the NH N atoms stems from the solvent (Scheme 3). Complex **4** was more conveniently purified after anion exchange with Na(BAr_4_) to give the corresponding BAr_4_^–^ salt (**4-BAr**_**4**_; see the SI). Noticeably, the latter salt was also obtained (along with other, yet uncharacterized, species) when using [H(OEt_2_)_2_](BAr_4_) instead of (NH_4_)PF_6_ in the protonation step following the low-temperature reduction of **1**. All of the above leave the nitrosyl ligands as the only possible source of the second N atom of the amidinate ligand present in the cation **4**.

Crystals of **4-PF**_**6**_ were of poor quality for diffraction purposes but still allowed for an unambiguous determination of the structure of the cation **4**, with two MoCp fragments symmetrically bridged by P^*t*^Bu_2_, NO, and acetamidinate ligands (Figure S1). This structure actually is comparable to that of the isoelectronic benzoate complex [Mo_2_Cp_2_(μ-PCy_2_)(μ-κ*O*:κ*O*′-O_2_CPh)(μ-CPh)](BAr_4_).^19^ Both of the above cations are 30-electron complexes for which a triple intermetallic bond must be proposed according to the 18-electron rule, which is consistent with the short intermetallic distance of 2.604(2) Å for **4-PF**_**6**_, only marginally longer than the one in the benzoate complex [2.576(1) Å], a difference likely due to the higher steric demands of the P^*t*^Bu_2_ ligand (vs PCy_2_). Spectroscopic indication for the presence of an acetamidinate ligand in **4-PF**_**6**_ is given by the observation of ^1^H NMR resonances at 2.27 ppm (3H, Me) and 9.43 ppm (br, 2H, NH); the NH groups also give rise to a stretch in the IR spectrum (Nujol mull) at 3366 cm^–1^, while the N–O stretch of the bridging nitrosyl appears at 1518 cm^–1^.

The fact that the formation of **4** is maximized when performing the reduction step of **1** at lower temperatures and shorter reaction times suggests that **4** stems from radical **A** (possibly isomer **A2**) formed after the first electron transfer (Scheme 3). Then a complex sequence of events, such as protonation (perhaps at a nitrosyl ligand),^2^ H-atom abstraction (likely at the N atom of the MeCN molecule to give an iminoacyl ligand), O-transfer (with unknown destination), and N–C reductive coupling between nitrene (NH) and iminoacyl (HN=CMe) ligands,^9a^ is likely in operation to build the amidinate ligand present in **4**, but the exact sequence of these elementary steps is unknown to date. Further experiments using other nitriles and reducing reagents are now underway to gain complementary information concerning the transformations described above and to evaluate their scope.

In summary, we have shown that several unusual transformations take place at the dimetal site of nitrosyl complex **1** upon reduction with Na(Hg) in MeCN under mild conditions. Two-electron reduction promotes an unprecedented full cleavage of the strong C≡N bond of a bridging MeCN molecule at room temperature to render an anionic vinylidene complex, which upon protonation yields the corresponding ethylidyne derivative in a selective and reversible way. In contrast, one-electron reduction of **1** and subsequent protonation trigger a complex sequence of steps including cleavage of the strong N≡O bond of a nitrosyl ligand and a nitrene/iminoacyl coupling to eventually build a bridging acetamidinate ligand. Further experiments are now underway to gain more insight into these transformations taking place under such mild conditions.

## Supporting Information Available

Preparative procedures, spectroscopic, microanalytical, and crystallographic data, results of DFT calculations, and spectra for new compounds (PDF)
Cartesian coordinates for all computed species (XYZ)

## Supplementary Material

ic3c03697_si_001.pdf

ic3c03697_si_002.xyz
