# Supplementary material for: C≡N and N≡O Bond Cleavages of Acetonitrile and Nitrosyl Ligands at a Dimolybdenum Center to Render Ethylidyne and Acetamidinate Ligands
Source: Inorg Chem. 2024 Feb 2;63(7):3207–11. doi: 10.1021/acs.inorgchem.3c03697 (PMC10880054; doi:10.1021/acs.inorgchem.3c03697)
Supplement: Supplementary file 1 — ic3c03697_si_001.pdf [file ic3c03697_si_001.pdf]

Supporting Information

C≡N and N≡O Bond Cleavages of Acetonitrile and Nitrosyl Ligands at a Dimolybdenum Center to Render Ethylidyne and Acetamidinate Ligands

M. Angeles Alvarez, M. Esther García, Daniel García-Vivó*, Ana M. Guerra, and Miguel A. Ruiz[a],

Departamento de Química Orgánica e Inorgánica/IUQOEM, Universidad de Oviedo, E-33071 Oviedo, Spain.

*E-mail: garciavdaniel@uniovi.es (D.G.V.), mara@uniovi.es (M.A.R).

Experimental Section

General Procedures and Starting Materials. All manipulations and reactions were carried out under an argon (99.995%) atmosphere using standard Schlenk techniques. Solvents were purified according to literature procedures, and distilled prior to use.[1] Compound [Mo_2Cp_2(μ-Cl)(μ-P^tBu_2)$(CO)_2$] was prepared as described previously,[2] and all other reagents were obtained from the usual commercial suppliers and used as received, unless otherwise stated. Petroleum ether refers to that fraction distilling in the range 338-343 K. Filtrations were carried out through diatomaceous earth unless otherwise stated. Chromatographic separations were carried out using jacketed columns refrigerated by a closed 2-propanol circuit kept at the desired temperature with a cryostat. Commercial aluminum oxide (activity I, 70-290 mesh) was degassed under vacuum prior to use. The latter was mixed under argon with the appropriate amount of water to reach activity IV. IR stretching frequencies of CO and NO ligands were generally measured in solution (using CaF_2 windows), are referred to as ν(XO) and are given in wave numbers (cm^{-1}). Nuclear magnetic resonance (NMR) spectra were recorded at 295 K unless otherwise stated. Chemical shifts (δ) are given in ppm, relative to internal tetramethylsilane (^1H, ^{13}C), or external 85% aqueous H_3PO_4 solutions (^{31}P). Coupling constants (J) are given in hertz. The visible-UV spectrum of **3** was recorded on a 10^{-3} M solution of the complex in dichloromethane.

Preparation of *trans*-[Mo_2Cp_2(μ-Cl)(μ-P^tBu_2)$(NO)_2$] (1). Nitric oxide (5% in Ar) was gently bubbled through a stirred solution of [Mo_2Cp_2(μ-Cl)(μ-P^tBu_2)$(CO)_2$] (ca. 1.0 mmol, prepared in situ from 0.500 g -1.020 mmol- of dimer [$Mo_2Cp_2(CO)_6$]) in tetrahydrofuran (15 mL) for 45 min at 233 K, to give a dark orange solution containing the monocarbonyl complex [Mo_2Cp_2Cl(μ-P^tBu_2)(CO)$(NO)_2$] as major product. After removal of the solvent under vacuum, the residue was dissolved in toluene (15 min) and the solution was refluxed for 15 min to give a yellow-brown solution. The solvent was afterwards removed, the residue was extracted with dichloromethane/petroleum ether (1/2), and the extracts were chromatographed on alumina at 288 K. Elution with the same solvent mixture gave a yellow fraction yielding, upon removal of solvents, compound **1** as a yellow microcrystalline solid (0.360 g, 63%). Anal. Calcd for $C_{18}H_{28}ClMo_2N_2O_2P$: C, 38.42; H, 5.02; N, 4.98. Found: C, 38.19; H, 5.76; N, 4.96. ν(NO) (CH_2Cl_2): 1602 (w, sh), 1575 (vs). $^{31}P\{^1H\}$ NMR (121.51 MHz, CD_2Cl_2): δ 281.8 (s, μ-P). ^1H NMR (400.13 MHz, CD_2Cl_2): δ 5.77 (s, 10H, Cp), 1.38 (d, J_{HP} = 14, 18H, ^tBu). $^{13}C\{^1H\}$ NMR (100.63 MHz, CD_2Cl_2): δ 97.0 (s, Cp), 45.2 [d, J_{CP} = 10, C^1(^tBu)], 33.7 (d, J_{CP} = 4, C^2(^tBu)].

Preparation of acetonitrile solutions of Na[Mo_2Cp_2(μ-P^tBu_2)(μ-CCH_2)$(NO)_2$] (2-Na). A solution of compound **1** (0.050 g, 0.089 mmol) in acetonitrile (10 mL) was stirred with excess Na(Hg) (ca. 0.5 ml of a 0.5% amalgam) for 40 min at room temperature, to give a yellow-

brown solution which was filtered using a canula. The resulting solution contained **2-Na** as major product, and was used without further purification. $^{31}P\{^1H\}$ RMN (121.5 MHz, MeCN-d_3): δ 229.8 (s, μ-P). ^1H NMR (300.13 MHz, MeCN-d_3): δ 6.87 (s, 2H, CH_2), 5.35 (s, 10H, Cp), 1.21 (d, J_{HP} = 12, 18H, ^{t}Bu).

Preparation of (DBUH)[Mo_2Cp_2(μ-P^tBu_2)(μ-CCH_2)$(NO)_2$] (2-DBUH). Neat 1,8-diazabicyclo[5.4.0]undec-7-ene, (DBU, 12μL, 0.080 mmol) was added to an acetonitrile solution (8 mL) of compound **3** (0.020 g, 0.036 mmol), and the mixture was stirred for 5 min to give a orange solution. After removal of the solvent, the residue was washed with petroleum ether (2 x 5 mL), and dried under vacuum to give compound **2-DBUH** as a yellow, air-sensitive solid. $^{31}P\{^1H\}$ RMN (121.5 MHz, MeCN): δ 230.5 (s, μ-P). ^1H NMR (400.13 MHz, MeCN-d_3, 238 K): δ 6.88 (s, 2H, CH_2), 5.50 (br, 1H, NH), 5.35 (s, 10H, Cp), 3.27 (m, 4H, CH_2), 3.12, 2.40, 1.77 (3m, 3 x 2H, CH_2), 1.68-1.52 (m, 6H, CH_2), 1.20 (d, J_{HP} = 12, 18H, ^{t}Bu). $^{13}C\{^1H\}$ NMR (100.64, MeCN-d_3, 238 K): δ 275.5 (s, μ-C), 162.9 (s, CN), 124.2 (s, μ-C*CH*$_2$), 94.8 (s, Cp), 52.6, 47.8, 40.9, 34.2, 29.2, 27.4, 25.1, 21.0 (8s, CH_2), 39.3 [d, J_{CP} = 7, C^1(^{t}Bu)], 33.1 [d, J_{CP} = 5, C^2(^{t}Bu)].

Preparation of [Mo_2Cp_2(μ-P^tBu_2)(μ-CMe)$(NO)_2$] (3). Solid NH_4PF_6 (0.021 g, 0.129 mmol) was added to a filtered solution of **2-Na** prepared as described before from compound **1** (0.050 g, 0.089 mmol), and the mixture was stirred at room temperature to give a dark purple solution. After removal of the solvent under vacuum, the residue was extracted with toluene, the extract was filtered, and the solvent removed again to give a purple residue that was dissolved in dichloromethane/petroleum ether (1/1) and chromatographed on alumina at 253 K. Elution with the same solvent mixture gave a purple fraction yielding, after removal of solvents, compound **3** as a purple microcrystalline solid (0.017 g, 34%). The crystals used in the X-ray study were grown by the slow diffusion of a layer of petroleum ether into a concentrated toluene solution of the complex at 253 K. Anal. Calcd for $C_{20}H_{31}Mo_2N_2O_2P$: C, 43.33; H, 5.64; N, 5.05. Found: C, 43.07; H, 5.49; N, 4.60. ν(NO) (CH_2Cl_2): 1598 (w, sh), 1566 (vs). $^{31}P\{^1H\}$ NMR (121.51 MHz, CD_2Cl_2): δ 236.7 (s, μ-P). ^1H NMR (400.13 MHz, CD_2Cl_2): δ 5.74 (s, 10H, Cp), 4.17 (d, J_{HP} = 2, 3H, Me), 1.25 (d, J_{HP} = 14, 18H, ^{t}Bu). $^{13}C\{^1H\}$ NMR (100.64 MHz, CD_2Cl_2): δ 487.8 (d, J_{CP} = 11, μ-C), 98.2 (s, Cp), 56.5 (s, CH_3), 41.3 [d, J_{CP} = 8, C^1(^{t}Bu)], 33.0 [d, J_{CP} = 5, C^2(^{t}Bu)]. Vis-UV (CH_2Cl_2): 540 nm (br, ε = 1980 L/cm.mol).

Preparation of [Mo_2Cp_2(μ-P^tBu_2)(μ-κN:$\kappa N'$-HNCMeNH)(μ-NO)]PF_6 (4-PF_6). A solution of compound **1** (0.030 g, 0.053 mmol) in acetonitrile (10 mL) was stirred with excess Na(Hg) (ca. 0.5 ml of a 0.5% amalgam) for 15 min at 273 K, to give a green solution which was filtered using a canula. Solid NH_4PF_6 (0.036 g, 0.221 mmol) was added to the filtered solution at 243 K, and the mixture was stirred at that temperature for 5 min to give a green solution. Removal of the solvent under vacuum gave a residue that was washed with toluene

(2 x 5 mL). The green resulting residue was then extracted with dichloromethane and filtered. Removal of the solvent from the filtrate gave compound **4-PF$_6$** as a green solid (0.015 g, 42%), contaminated with small amounts of other unidentified species. Full purification of **4-PF$_6$** could only be achieved by the slow diffusion of a layer of petroleum ether into a concentrated tetrahydrofuran solution of the complex. This yielded small amounts of X-ray quality crystals of pure **4-PF$_6$**. Anal. Calcd for $C_{20}H_{33}F_6Mo_2N_3OP_2$: C, 34.35; H, 4.76; N, 6.01. Found: C, 34.06; H, 4.35; N, 5.72. ν(NO) (CH_2Cl_2): 1517 (s). ν(NX) (Nujol): 3366 (s, NH), 1518 (s, NO). $^{31}P\{^1H\}$ NMR (121.51 MHz, CD_2Cl_2): δ 339.6 (s, μ-P). ^1H NMR (300.13 MHz, CD_2Cl_2): δ 9.43 (s, br, 2H, NH), 6.16 (s, 10H, Cp), 2.29 (s, 3H, CH_3), 1.21 (d, J_{HP} = 15, 9H, ^{t}Bu), 0.90 (d, br, J_{HP} = 14, 9H, ^{t}Bu).

Preparation of [Mo$_2$Cp$_2$(μ-P^{t}Bu$_2$)(μ-κN:$\kappa N'$-HNCMeNH)(μ-NO)]BAr$_4$ (4-BAr$_4$). A solution of crude compound **4-PF$_6$** (0.030 g, ca. 0.043 mmol) in dichloromethane (8 mL) was stirred with Na(BAr$_4$) (Ar = 3,5-$C_6H_3(CF_3)_2$, 0.045 g, 0.050 mmol) for 5 min at room temperature. After removal of the solvent, the residue was extracted with dichloromethane/petroleum ether (2/1) and the extracts were chromatographed on alumina at 253 K. Elution with dichloromethane/petroleum ether (3/1) gave a green fraction yielding, upon removal of solvent, compound **4-BAr$_4$** as a dark-green microcrystalline powder (0.025 g, 41%). Anal. Calcd for $C_{52}H_{45}BF_{24}Mo_2N_3OP$: C, 44.06; H, 3.20; N, 2.96. Found: C, 43.76; H, 3.43; N, 2.90. ν(NO) (CH_2Cl_2): 1510 (s). $^{31}P\{^1H\}$ NMR (121.51 MHz, CD_2Cl_2): δ 342.8 (s, μ-P). ^1H NMR (400.13 MHz, CD_2Cl_2): δ 9.00 (s, br, 2H, NH), 7.72 (s, br, 8H, C_6H_3), 7.56 (s, 4H, C_6H_3), 6.13 (s, 10H, Cp), 2.25 (s, 3H, CH_3), 1.18 (d, J_{HP} = 15, 9H, ^{t}Bu), 0.93 (d, br, J_{HP} = 14, 9H, ^{t}Bu). $^{13}C\{^1H\}$ NMR (100.64 MHz, CD_2Cl_2): δ 177.2 (s, CN_2), 162.2 [q, J_{C11B} = 49, $C^1(C_6H_3)$], 135.2 [s, $C^2(C_6H_3)$], 129.3 [qq, J_{CF} = 31, J_{C11B} =3, $C^3(C_6H_3)$], 124.9 (q, J_{CF} = 273, CF_3), 117.9 [hp, J_{CF} = 4, $C^4(C_6H_3)$], 103.4 (s, Cp), 46.7 [d, J_{CP} = 16, C^1(^{t}Bu)], 45.2 [d, J_{CP} = 3, C^1(^{t}Bu)], 33.8 [s, br, C^2(^{t}Bu)], 31.6 [d, J_{CP} = 5, C^2(^{t}Bu)], 27.1 (s, CH_3).

X-Ray Structure Determination of Compound 3. Data collection for this compound was performed at 100 K on a Bruker D8 Venture Photon III 14 κ-geometry diffractometer, using MoK$_\alpha$ radiation. The software *APEX4*[3] was used for collecting frames with the ω/ϕ scan measurement method. The *SAINT V8.40B* software was used for data reduction,[4] and a multi-scan absorption correction was applied with *SADABS-2016/2*.[5] Using the program suite *WinGX*,[6] the structure was solved by Patterson interpretation and phase expansion using *SHELXL2018/3*, and refined with full-matrix least squares on F^2 using *SHELXL2018/3*.[7] All non-hydrogen atoms were generally refined anisotropically, except those involved in disorder, and all hydrogen atoms were geometrically placed and refined using a riding model to give the residuals shown in Table S1, except for the H atoms of the methyl group, found to be disordered over two rotationally-related sites, which were satisfactorily refined with 0.5/0.5 occupancies.

X-Ray Structure Determination of Compound 4-PF_6. Data collection for this compound was performed at 150 K on an Oxford Diffraction Xcalibur Nova single crystal diffractometer, using Cu Kα radiation. Images were collected at a 62 mm fixed crystal-detector distance using the oscillation method, with 1.30º oscillation and variable exposure time per image (30-100 s). Data collection strategy was calculated with the program *CrysAlis Pro CCD*,[8] and data reduction and cell refinement were performed with the program *CrysAlis Pro RED*.[8] An empirical absorption correction was applied using the SCALE3 ABSPACK algorithm as implemented in the program *CrysAlis Pro RED*, and structure solution and refinements were performed as described for compound **3**. In the case of **4-PF_6**, two independent cations and anions were present in the unit cell, similar to each other. One of the cations displayed one Cp ring disordered over two sites, satisfactorily refined with 0.5/0.5 occupancies. The second cation displayed disorder at both Cp ligands and the ^{t}Bu groups, but only the disorder of the Cp ligands could be satisfactorily modeled, also over two sites with 0.5/0.5 occupancies. The N-bound H atoms were located on difference maps and were refined riding on their parent atoms, but a restraint on the N–H bond length (to 1.02 ± 0.02) Å) was necessary to obtain a satisfactory refinement of their positions. Because of the poor quality of the crystal used in the study, a significant number of residual peaks of electron density (in the range 1-3 electrons) remained in the final difference map. This accounts for the modest agreement parameters of this structural study (R_1 = 0.14; wR_2 = 0.40; GOF = 1.673), even if the structure of the cations and anions in the unit cell are well defined.

Computational Details. DFT calculations on compounds **2**, **3** and intermediates **A1**, **A2** and **B** were carried out using the GAUSSIAN16 package,[9] and the M06L functional.[10] A pruned numerical integration grid (99,590) was used for all the calculations *via* the keyword Int=Ultrafine together with the empirical dispersion correction from Grimme and co-workers via the keyword GD3.[11] Effective core potentials and their associated double-ζ LANL2DZ basis set were used for Mo atoms.[12] The light elements (P, N, O, C and H) were described with the 6-31G* basis.[13] Geometry optimizations were performed under no symmetry restrictions, using initial coordinates derived from the closest X-ray data available. Frequency analyses were performed for all the stationary points to ensure that a minimum structure with no imaginary frequencies was achieved. The effect of solvent (acetonitrile) on the stability of isomers **A1** and **A2** in solution was modeled through the polarized-continuum-model (PCM) of Tomasi and co-workers,[14] using Truhlar and co-workers SMD solvation model[15] on the gas-phase optimized structures. The lowest 50 singlet-singlet excitations for complex **3** were computed by means of TD-DFT calculations at the ground state singlet optimized geometry in order to simulate its UV–Vis absorption spectra. The results from these calculations were analyzed with the software Chemissian (v4.6).

References

1. Armarego, W. L. F.; Chai, C. *Purification of Laboratory Chemicals, 7th ed.*; Butterworth-Heinemann: Oxford, U. K, 2012.
2. Alvarez, M. A.; Casado-Ruano, M.; García, M. E.; García-Vivó, D.; Ruiz, M. A. Structural and chemical effects of the $P^{t}Bu_2$ bridge at unsaturated dimolybdenum complexes having hydride and hydrocarbyl ligands. *Inorg. Chem.* **2017**, *56*, 11336-11351.
3. *APEX4 v2021.10-0*; Bruker AXS Inc.: Madison (WI), USA, 2021.
4. *SAINT v8.40B*; Bruker AXS Inc.: Madison (WI), USA, 2018.
5. Krause, L.; Herbst-Irmer, R.; Sheldrick G.M.; Stalke D. Comparison of silver and molybdenum microfocus X-ray sources for single-crystal structure determination *J. Appl. Cryst.* **2015**, *48*, 3-10.
6. Farrugia, L. J. WinGX suite for small-molecule single-crystal crystallography. *J. Appl. Cryst.* **1999**, *32*, 837-838.
7. (a) Sheldrick, G. M. *SHELXL2018*; University of Gottingen, Germany, 2018. (b) Sheldrick, G. M. Crystal structure refinement with SHELXL. *Acta Crystallogr. Sect. C* **2015**, *71*, 3-8. (c) Sheldrick, G. M. A short history of SHELX. *Acta Crystallogr. Sect. A* **2008**, *64*, 112-122.
8. *CrysAlis Pro*; Oxford Diffraction Limited, Ltd.: Oxford, U. K., 2006.
9. Frisch, M. J.; Trucks, G. W.; Schlegel, H. B.; Scuseria, G. E.; Robb, M. A.; Cheeseman, J. R.; Scalmani, G.; Barone, V.; Petersson, G. A.; Nakatsuji, H.; Li, X.; Caricato, M.; Marenich, A. V.; Bloino, J.; Janesko, B. G.; Gomperts, R.; Mennucci, B.; Hratchian, H. P.; Ortiz, J. V.; Izmaylov, A. F.; Sonnenberg, J. L.; Williams-Young, D.; Ding, F.; Lipparini, F.; Egidi, F.; Goings, J.; Peng, B.; Petrone, A.; Henderson, T.; Ranasinghe, D.; Zakrzewski, V. G.; Gao, J.; Rega, N.; Zheng, G.; Liang, W.; Hada, M.; Ehara, M.; Toyota, K.; Fukuda, R.; Hasegawa, J.; Ishida, M.; Nakajima, T.; Honda, Y.; Kitao, O.; Nakai, H.; Vreven, T.; Throssell, K.; Montgomery, J. A., Jr.; Peralta, J. E.; Ogliaro, F.; Bearpark, M. J.; Heyd, J. J.; Brothers, E. N.; Kudin, K. N.; Staroverov, V. N.; Keith, T. A.; Kobayashi, R.; Normand, J.; Raghavachari, K.; Rendell, A. P.; Burant, J. C.; Iyengar, S. S.; Tomasi, J.; Cossi, M.; Millam, J. M.; Klene, M.; Adamo, C.; Cammi, R.; Ochterski, J. W.; Martin, R. L.; Morokuma, K.; Farkas, O.; Foresman, J. B.; Fox, D. J. *Gaussian 16, Revision A.03*; Gaussian, Inc.: Wallingford, CT, USA, 2016.
10. Zhao Y.; Truhlar, D. G. A new local density functional for main-group thermochemistry, transition metal bonding, thermochemical kinetics, and noncovalent interactions. *J. Chem. Phys.* **2006**, *125*, 194101: 1-18.

11. Grimme, S.; Antony, J.; Ehrlich, S.; Krieg, H. A consistent and accurate ab initio parameterization of density functional dispersion correction (DFT-D) for the 94 elements H-Pu. *J. Chem. Phys.* **2010**, *132*, 154104.
12. Hay, P. J.; Wadt, W. R. Ab initio effective core potentials for molecular calculations. Potentials for potassium to gold including the outermost core orbitals. *J. Chem. Phys.* **1985**, *82*, 299-310.
13. (a) Hariharan, P. C.; Pople, J. A. Influence of polarization functions on MO hydrogenation energies. *Theor. Chim. Acta* **1973**, *28*, 213-222. (b) Petersson, G. A.; Al-Laham, M. A. A complete basis set model chemistry. II. Open-shell systems and the total energies of the first-row atoms. *J. Chem. Phys.* **1991**, *94*, 6081-6090. (c) Petersson, G. A.; Bennett, A.; Tensfeldt, T. G.; Al-Laham, M. A.; Shirley, W. A.; Mantzaris, J. A complete basis set model chemistry. I. The total energies of closed-shell atoms and hydrides of the first-row elements. *J. Chem. Phys.* **1988**, *89*, 2193-2218.
14. (a) Tomasi, J.; Mennucci, B.; Cammi, R. Quantum mechanical continuum solvation models. *Chem. Rev.* **2005**, *105*, 2999-3093. (b) Cossi, M.; Barone, V.; Cammi, R.; Tomasi, J. Ab initio study of solvated molecules: a new implementation of the polarizable continuum model. *Chem. Phys. Lett.* **1996**, *255*, 327-335, and references therein.
15. Marenich, A. V.; Cramer, C. J.; Truhlar, D. G. Universal solvation model based on solute electron density and a continuum model of the solvent defined by the bulk dielectric constant and atomic surface tensions, *J. Phys. Chem. B* **2009**, *113*, 6378-6396.

Table S1. Crystal Data for New Compounds

	**3**	**4-PF$_6$**
mol formula	$C_{20}H_{31}Mo_2N_2O_2P$	$C_{20}H_{33}F_6Mo_2N_3OP_2$
mol wt	554.32	699.31
cryst syst	monoclinic	monoclinic
space group	*C*2/*c*	*C*2/*c*
radiation (λ, Å)	0.71073	1.54184
a, Å	30.3541(11)	58.036(4)
b, Å	9.3985(3)	8.9232(6)
c, Å	15.8197(6)	20.8134(14)
α, deg	90	90
β, deg	102.1620(10)	99.877(7)
γ, deg	90	90
V, Å^3	4411.8(3)	10618.7(12)
Z	8	16
calcd density, g cm^{-3}	1.669	1.750
absorp coeff, mm^{-1}	1.226	9.431
temperature, K	100.0(1)	150(1)
θ range (deg)	2.27-32.58	4.31-70.60
index ranges (*h*, *k*, *l*)	–45, 45; –14, 11; –23, 23	–67, 70; –10, 7; –24, 24
no. of reflns collected	77161	23957
no. of indep reflns (R_{int})	8004 (0.0581)	9809 (0.0779)
reflns with $I > 2\sigma(I)$	6609	7471
R indexes [data with $I > 2\sigma(I)$]^a	R_1 = 0.0279; wR_2 = 0.0526 ^{b}	R_1 = 0.1408; wR_2 = 0.4059^c
R indexes (all data)^a	R_1 = 0.0412; wR_2 = 0.0560 ^{b}	R_1 = 0.1594; wR_2 = 0.4379^c
GOF	1.032	1.673
no. of restraints/params	0 / 244	15 / 556
$\Delta\rho$(max., min.), eÅ^{-3}	1.049 / –0.756	3.261 / –2.534
CCDC deposition no	2300630	2300631

^{a} $R = \Sigma||F_o| - |F_c|| / \Sigma|F_o|$. $wR = [\Sigma w(|F_o|^2 - |F_c|^2)^2 / \Sigma w|F_o|^2]^{1/2}$. $w = 1/[\sigma^2(F_o^2) + (aP)^2 + bP]$ where $P = (F_o^2 + 2F_c^2)/3$. ^{b} a = 0.0191, b = 6.2940. ^{c} a = 0.2000, b = 0.0000.

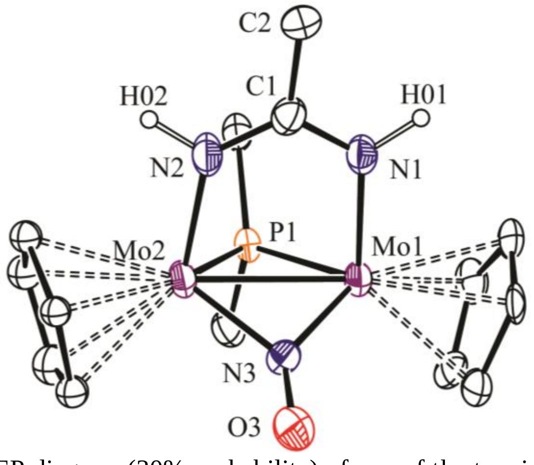

Figure S1. ORTEP diagram (30% probability) of one of the two independent molecules of the cation in compound **4-PF$_6$**, with ^{t}Bu groups (except their C^1 atoms) and most H atoms omitted. Selected bond lengths (Å): Mo1–Mo2 = 2.604(2); Mo1–P1 = 2.389(4); Mo1–N1 = 2.09(1); Mo1–N3 = 1.95(1); N1–C1 = 1.31(2).

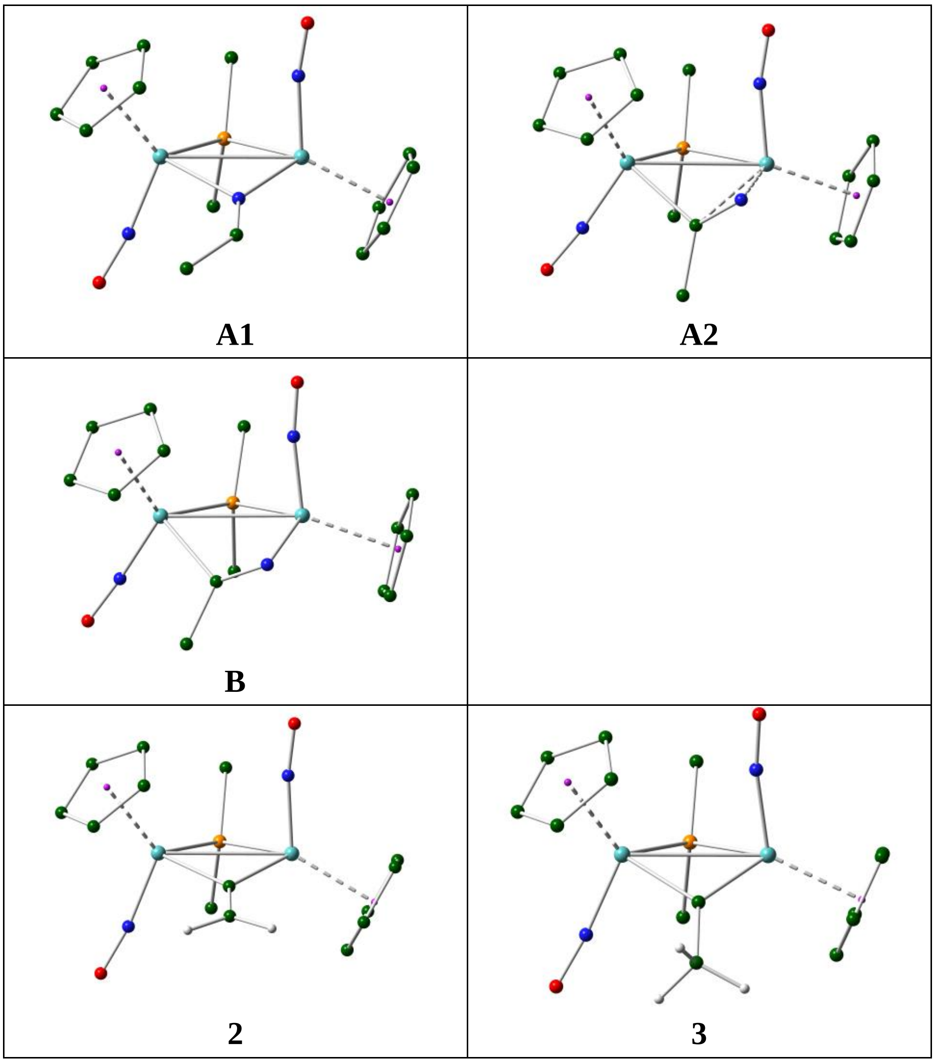

Figure S2. M06L-DFT-optimized structures of complexes **A1**, **A2**, **B**, **2** and **3**, with most H atoms and ^{t}Bu groups (except their C^1 atoms) omitted. The Gibbs free energy of isomer **A2** at 295 K was 10.0 kJ/mol higher than that of **A1** in the gas phase, but just 4.6 kJ/mol higher in acetonitrile solution.

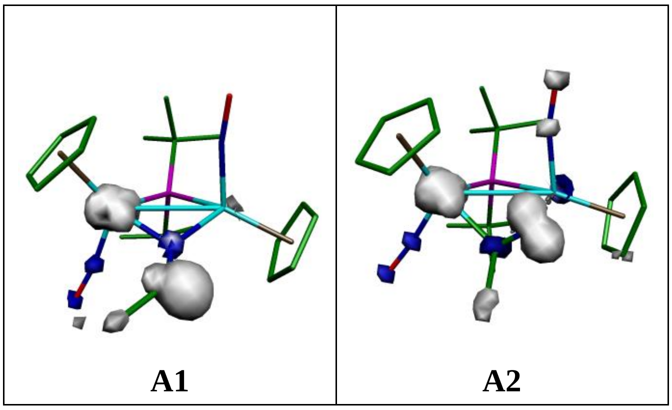

Figure S3. M06L-DFT-computed total spin density for isomers **A1** and **A2**. H atoms are omitted.

Table S2. Selected bond lengths (Å) and angles (°) for the M06L-DFT-optimized structures of complexes **A1**, **A2**, **B**, **2** and **3**.[a]

Parameter	**A1**	**A2**	**B**	**2**	**3**	**3** (exp)
Mo–Mo	2.916	2.985	3.037	2.929	2.921	2.9218(2)
Mo1–P	2.455	2.458	2.432	2.450	2.486	2.4833(5)
Mo1–N	1.795	1.790	1.788	1.792	1.800	1.771(2)
Mo1–C_b		2.158	2.189	2.111	2.032	2.028(2)
Mo1–N_b	2.197					
Mo2-P	2.460	2.497	2.455	2.449	2.487	2.4837(4)
Mo2–N	1.802	1.801	1.776	1.797	1.818	1.797(2)
Mo2–C_b		2.371	2.573	2.097	2.006	1.997(2)
Mo2–N_b	2.107	2.169	2.098			
Mo1–Mo2–N	86.31	82.44	84.65	86.14	81.38	82.15(5)
Mo2–Mo1–N	110.08	118.07	120.16	110.25	112.70	111.81(5)

[a] Mo1 and Mo2 refer to the metal atoms on the left and right at figure S1, respectively; a “b” subindex denotes metal-bound atoms of *bridging* ligands.

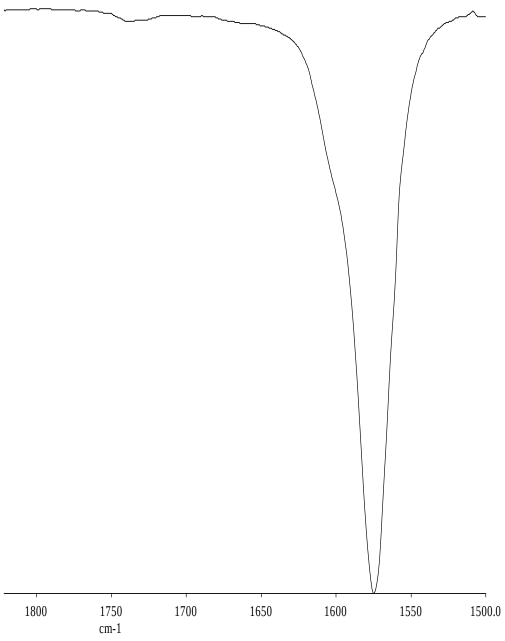

Figure S4. IR spectrum of compound **1** in dichloromethane solution.

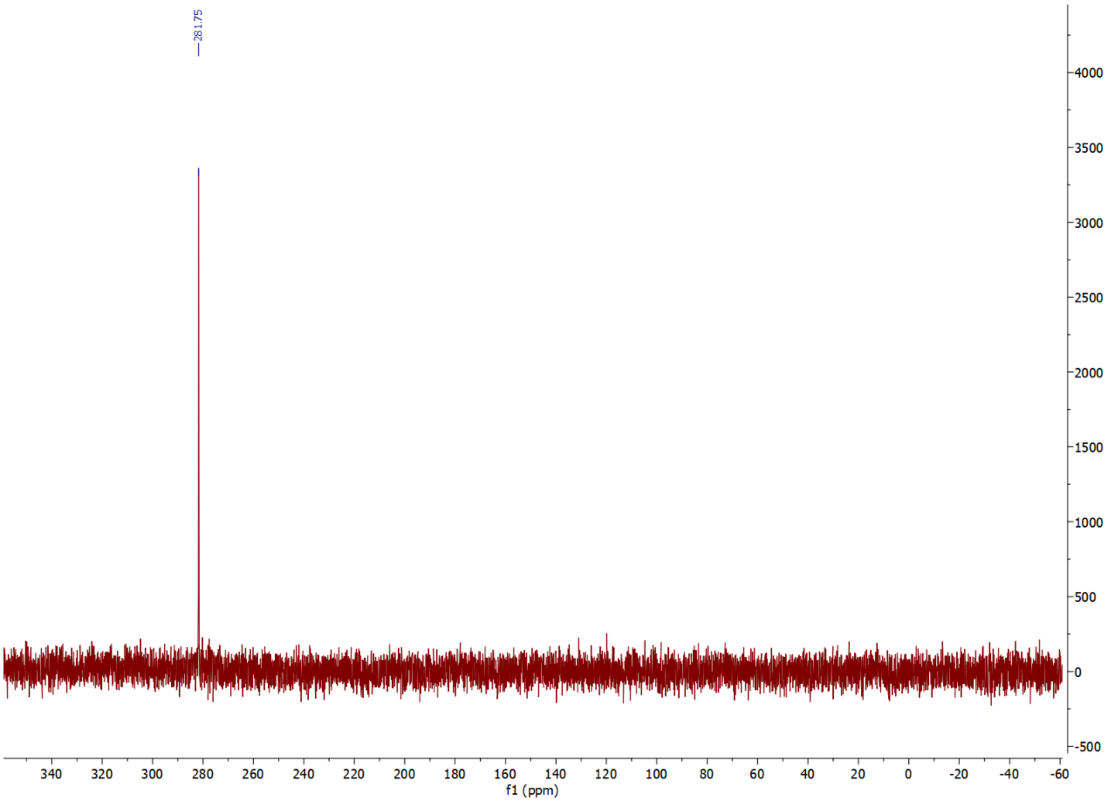

Figure S5. $^{31}P\{^1H\}$ NMR spectrum of compound **1**(CD_2Cl_2).

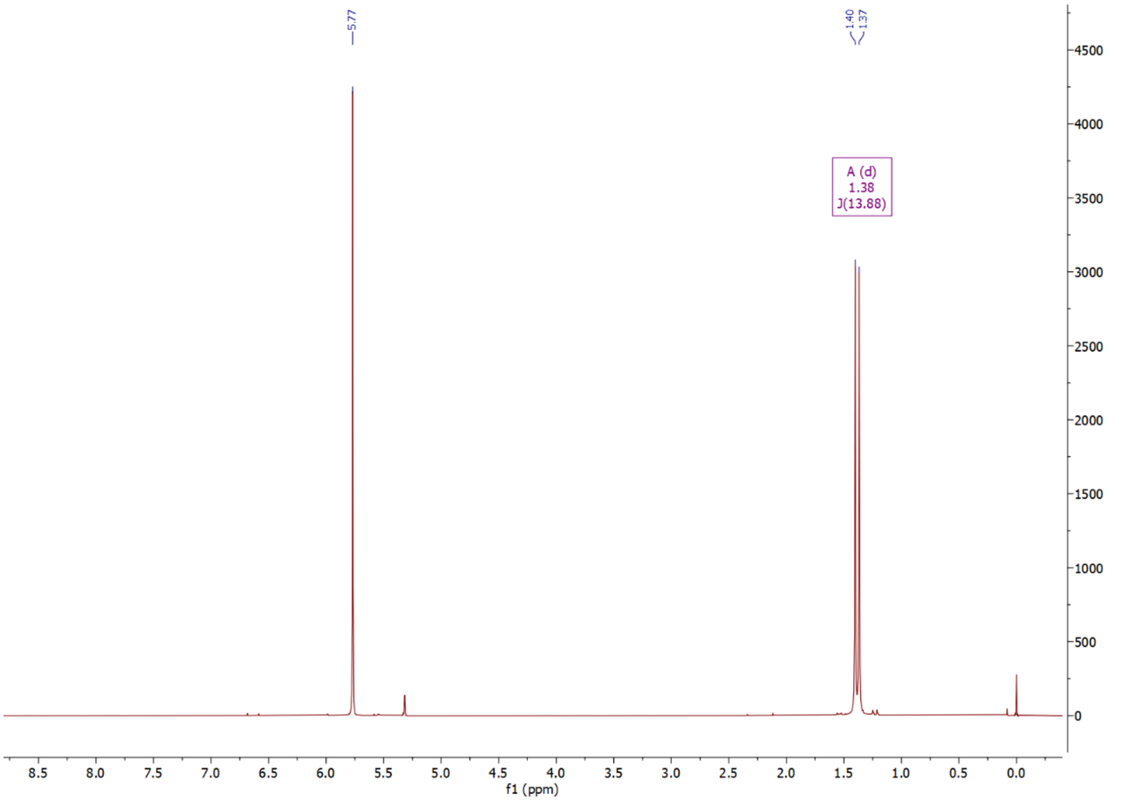

Figure S6. ^{1}H NMR spectrum of compound **1** (CD_2Cl_2).

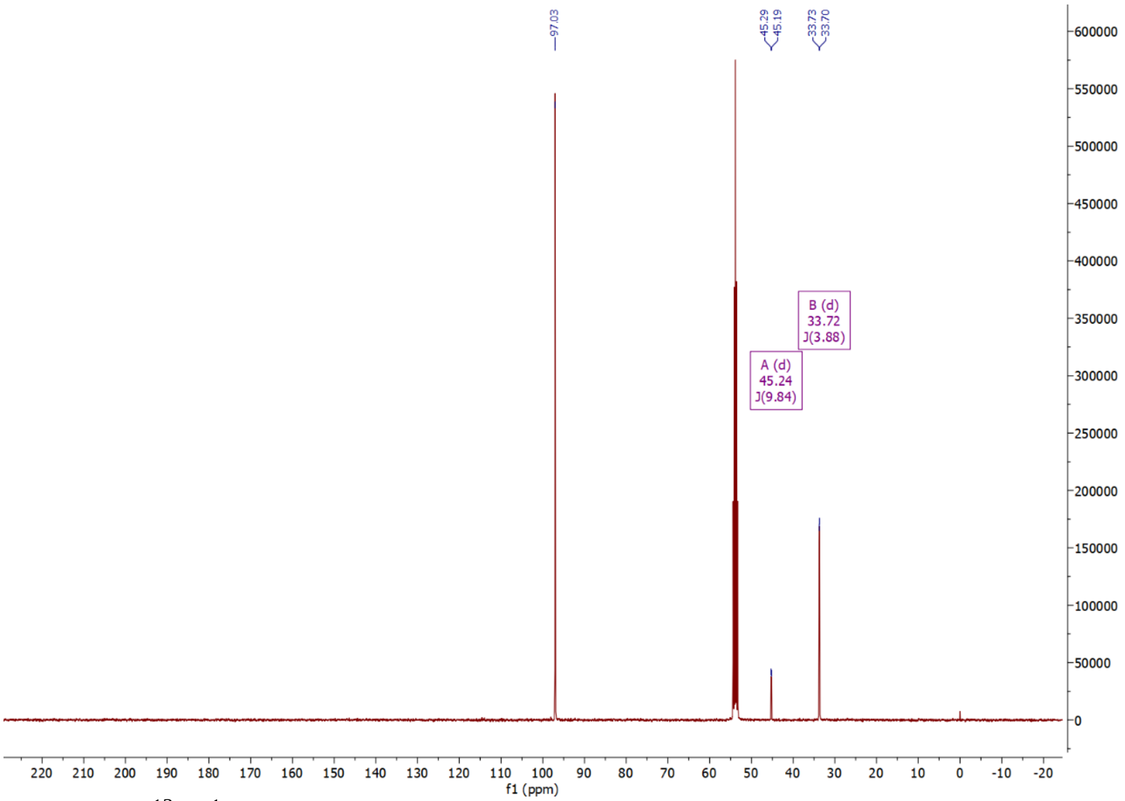

Figure S7. $^{13}C\{^{1}H\}$ NMR spectrum of compound **1** (CD_2Cl_2).

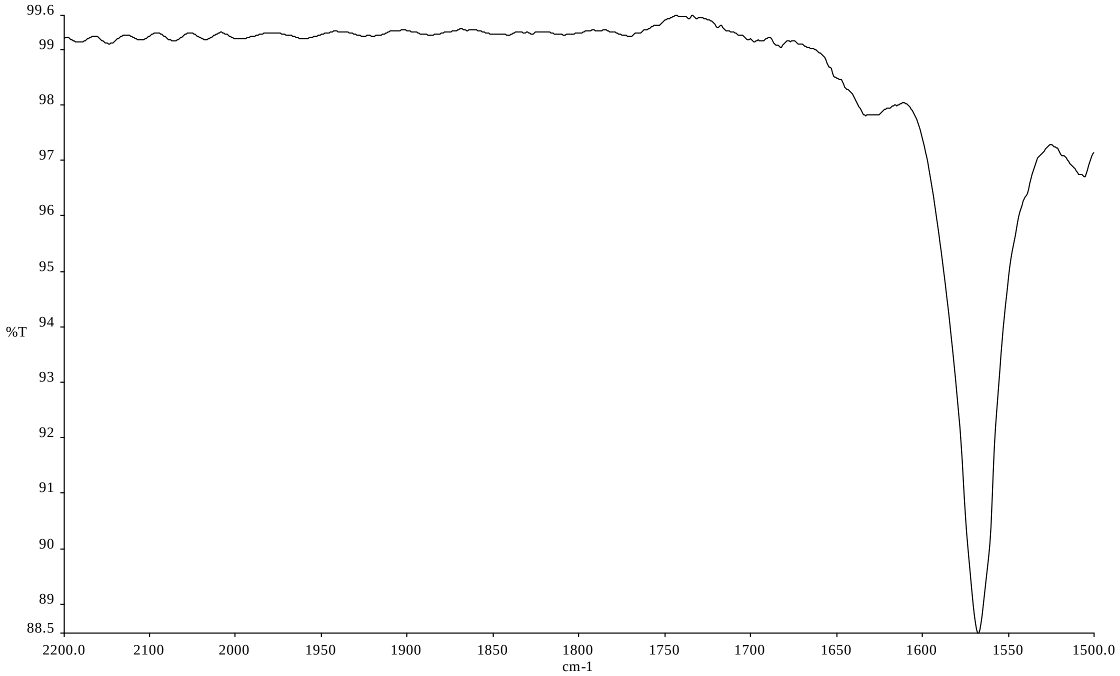

Figure S8. IR spectrum of the presumed intermediate **A** in acetonitrile solution.

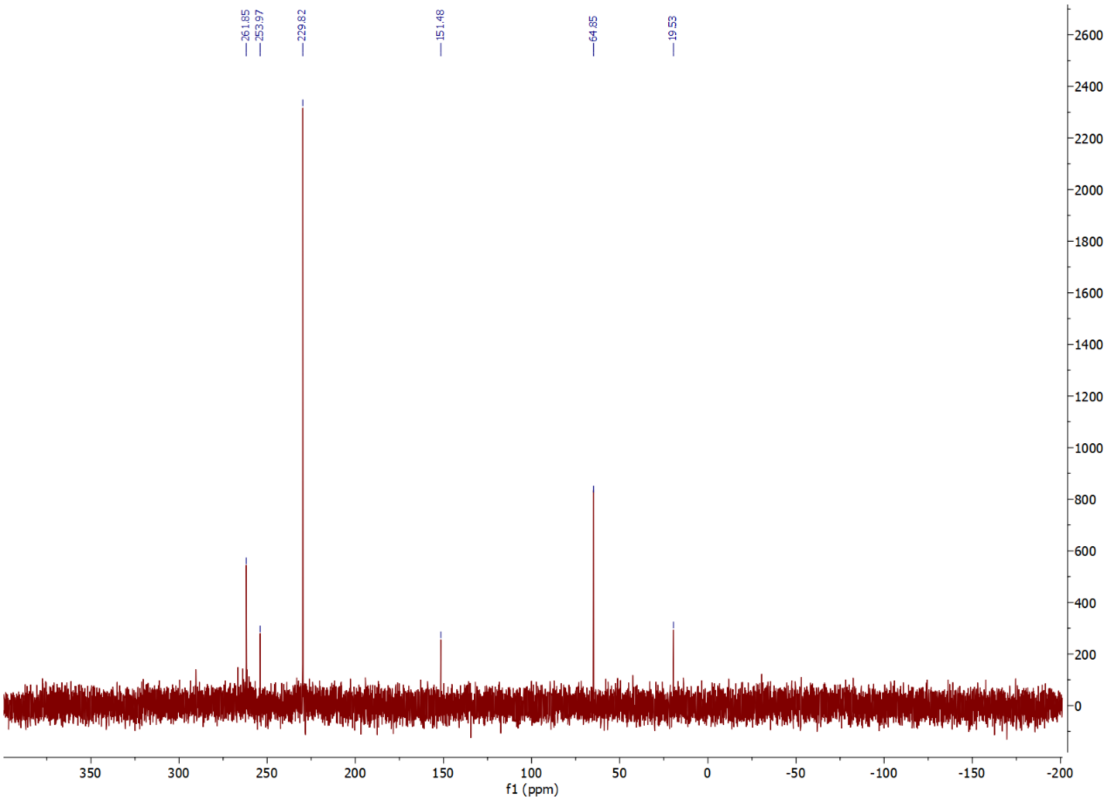

Figure S9. $^{31}P\{^{1}H\}$ NMR spectrum of a crude reaction mixture containing compound **2-Na** (δ 229.8 ppm) as major product (MeCN-d_3).

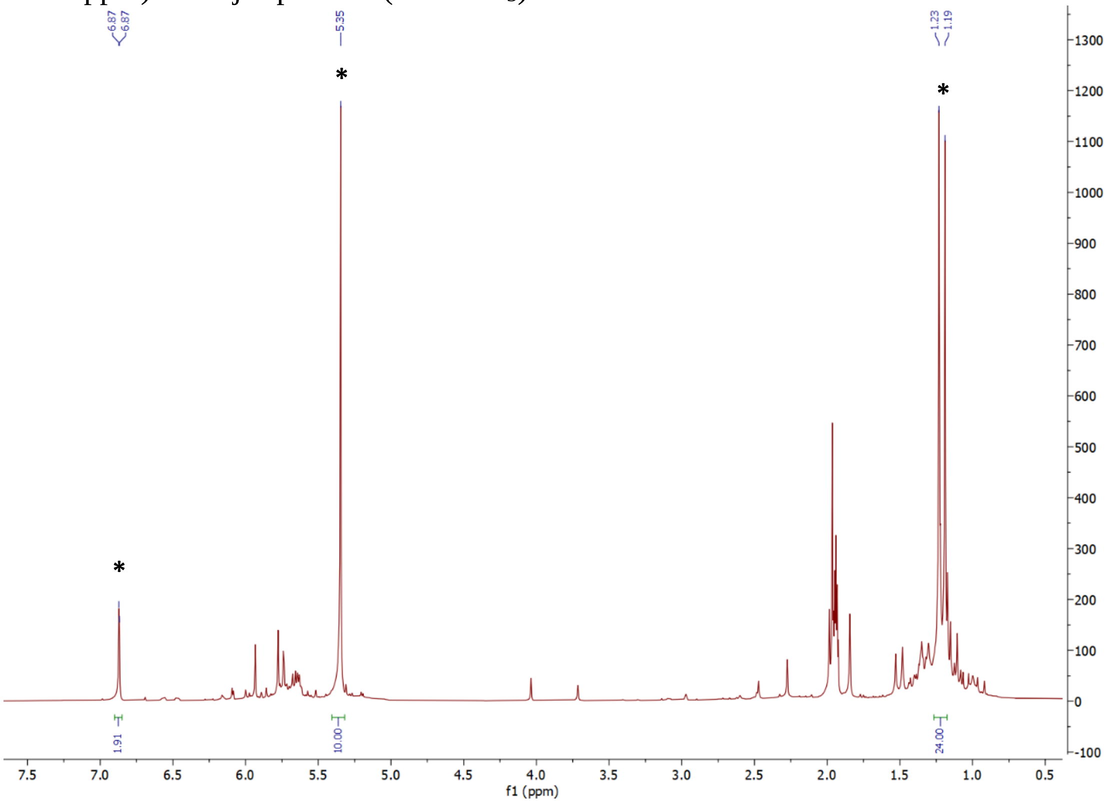

Figure S10. ^{1}H NMR spectrum of a crude reaction mixture containing compound **2-Na** (resonances marked *) as major product (MeCN-d_3).

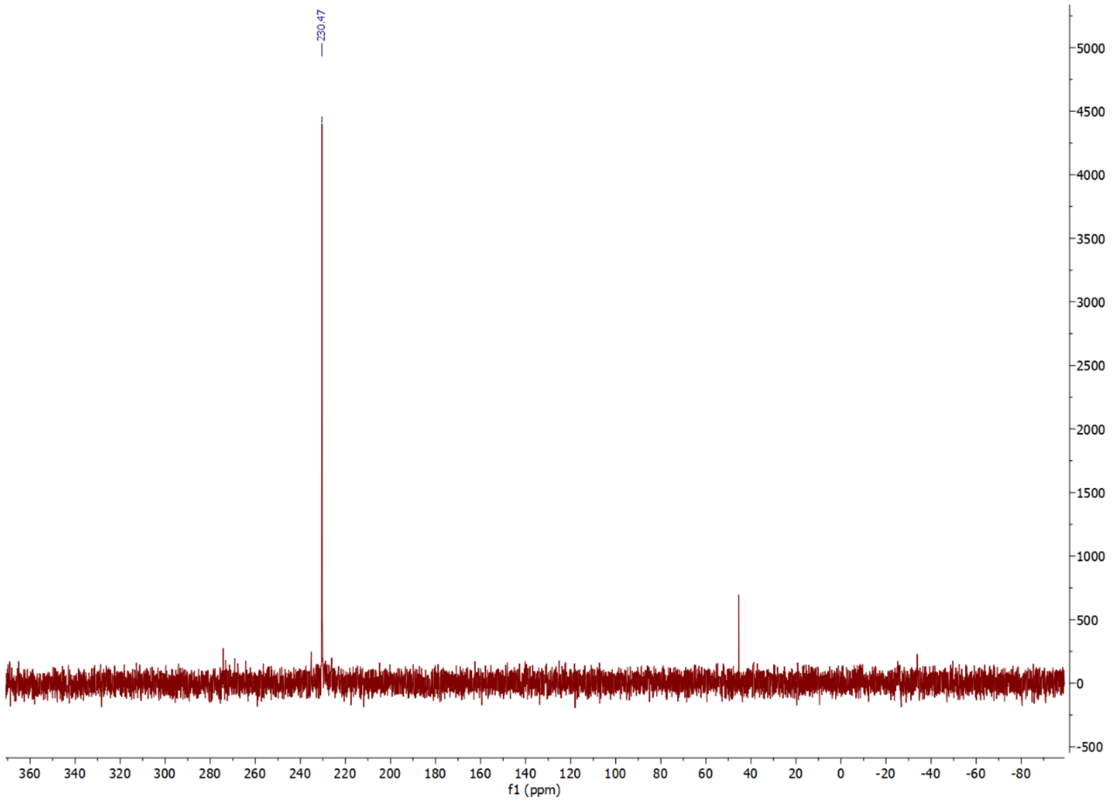

Figure S11. $^{31}P\{^{1}H\}$ NMR spectrum of compound **2-DBUH** (MeCN-d_3).

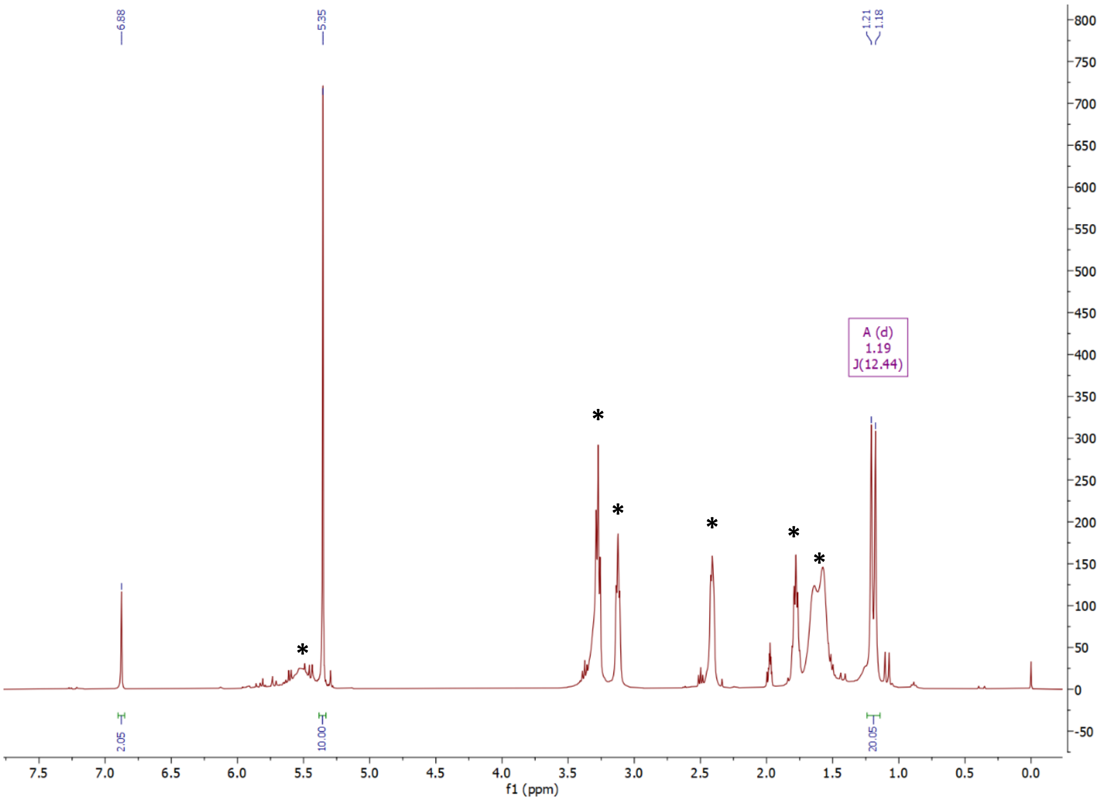

Figure S12. ^{1}H NMR spectrum of compound **2-DBUH** (MeCN-d_3); resonances marked with * correspond to the $DBUH^{+}$ cation.

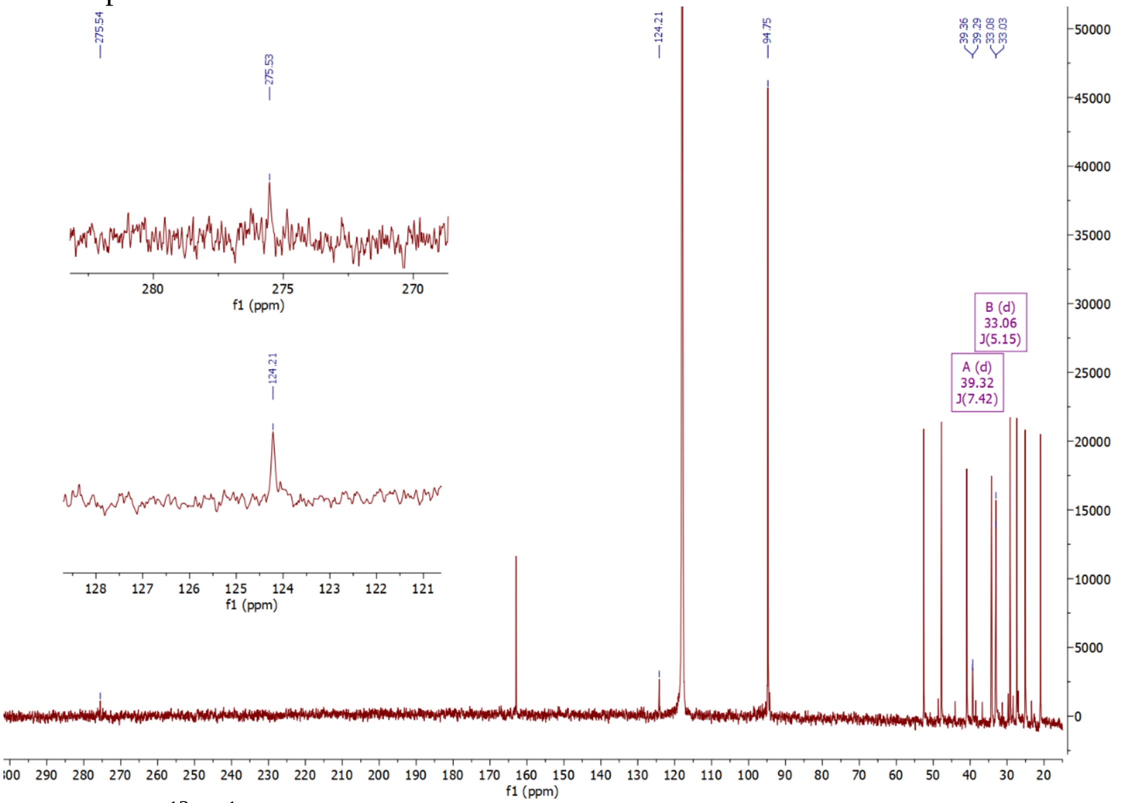

Figure S13. $^{13}C\{^{1}H\}$ NMR spectrum of compound **2-DBUH** (MeCN-d_3)

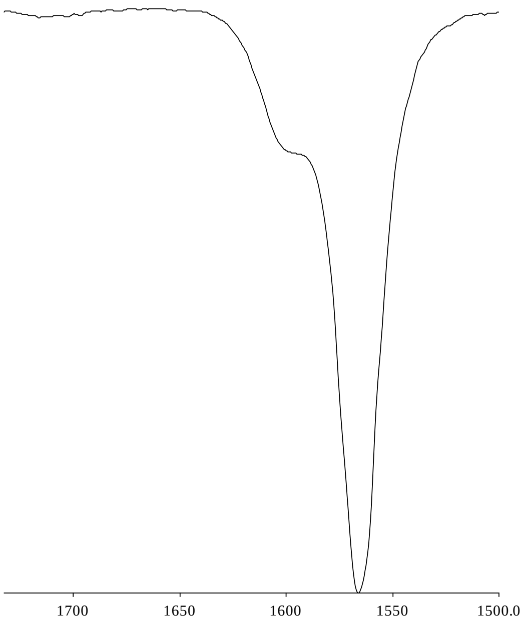

Figure S14. IR spectrum of compound **3** in dichloromethane solution.

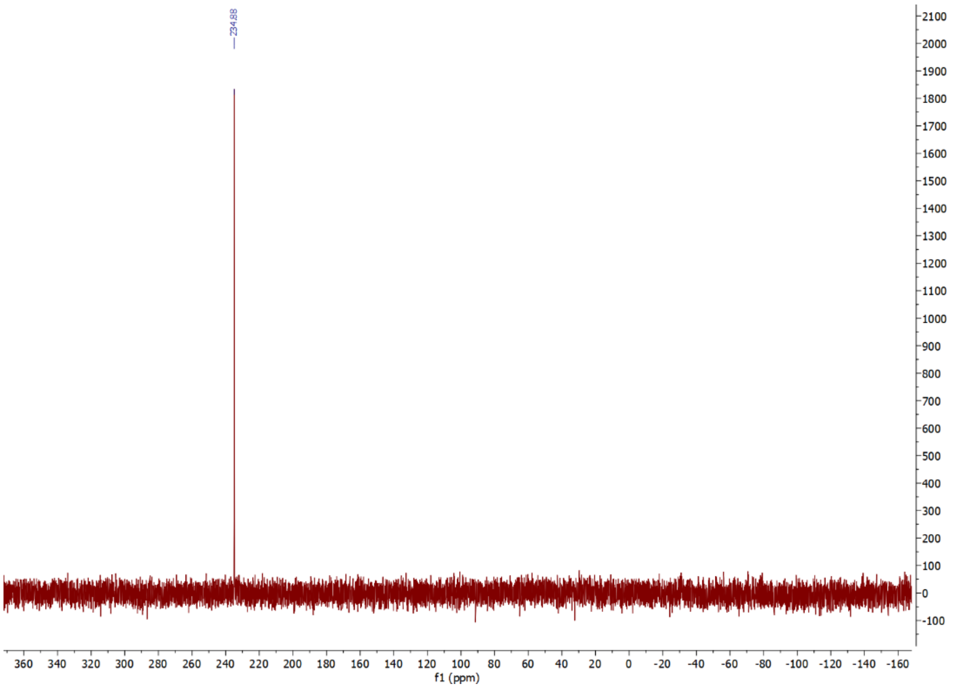

Figure S15. $^{31}P\{^{1}H\}$ NMR spectrum of compound **3** (C_6D_6).

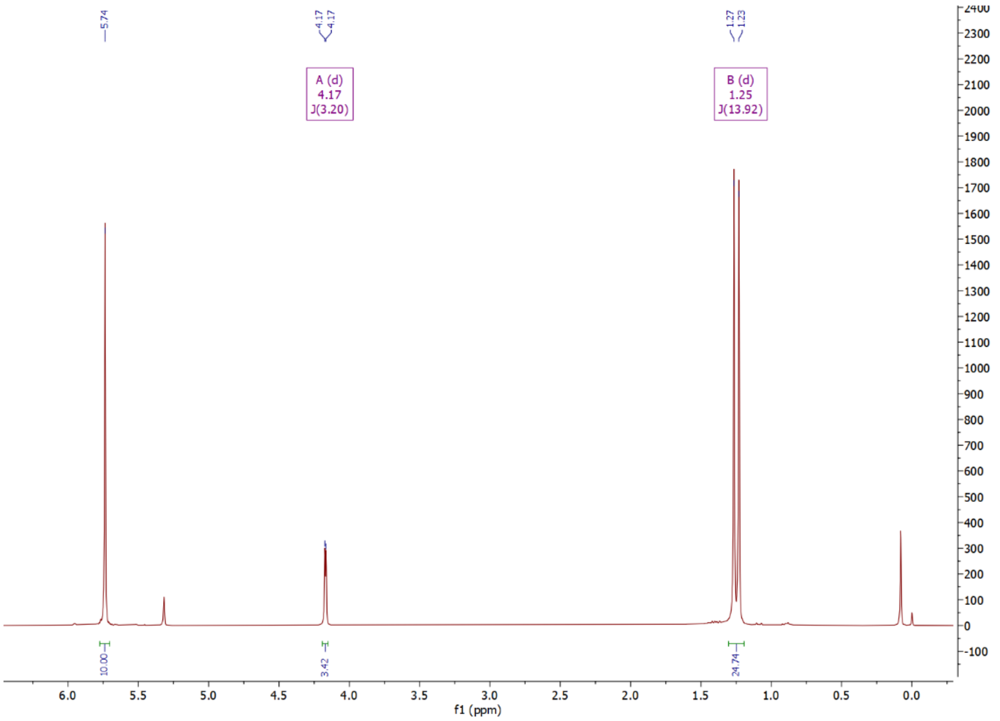

Figure S16. ^{1}H NMR spectrum of compound **3** (CD_2Cl_2).

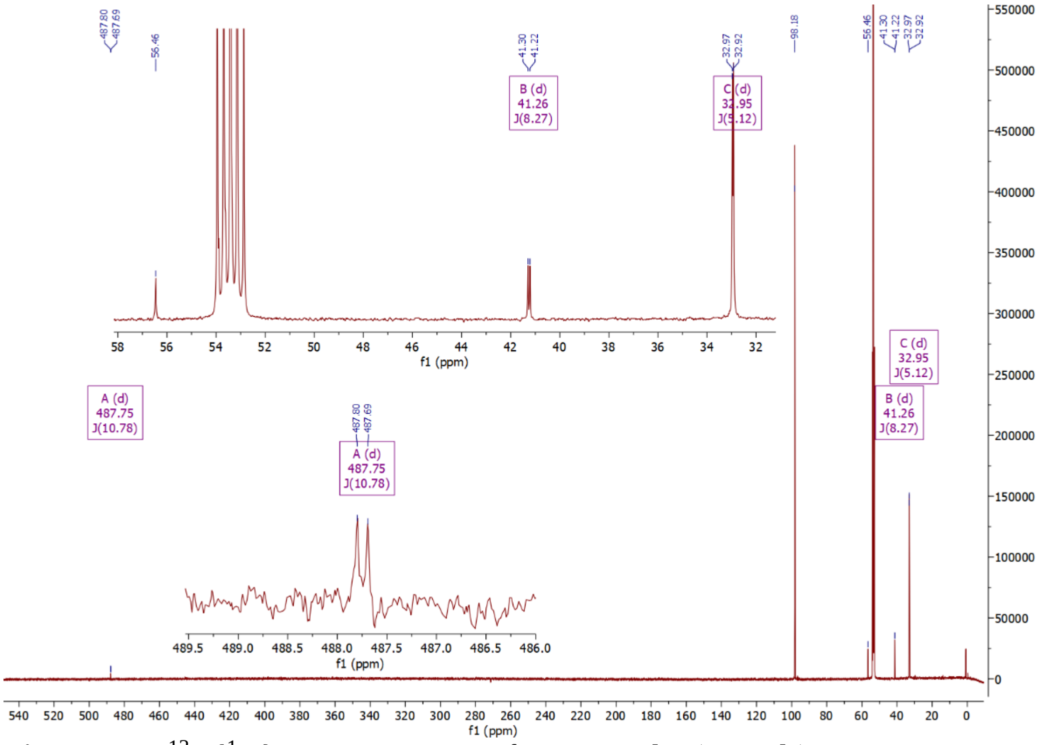

Figure S17. $^{13}C\{^{1}H\}$ NMR spectrum of compound **3** (CD_2Cl_2).

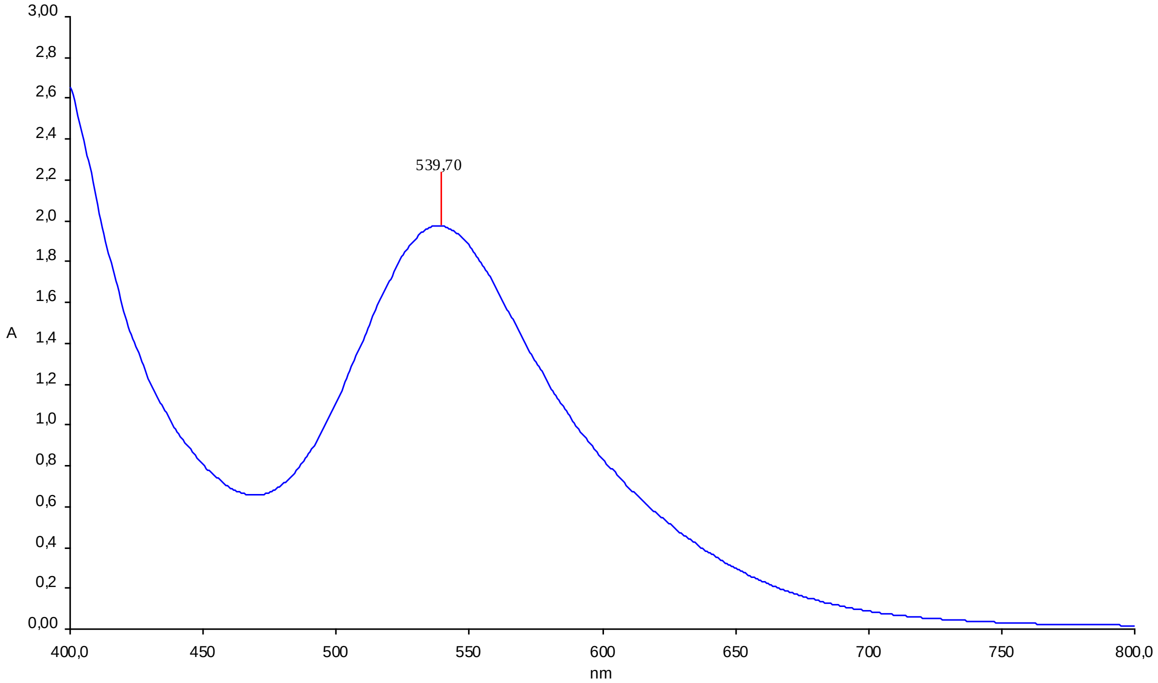

Figure S18. Visible spectrum of compound **3** (10^{-3} M in CH_2Cl_2).

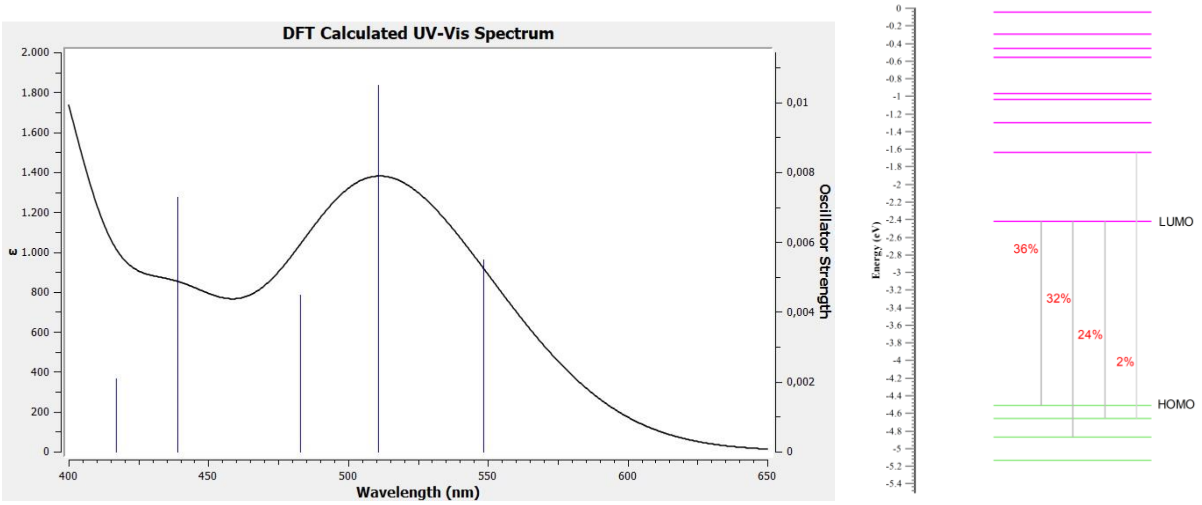

Figure S19. TD-DFT computed electronic spectrum of compound **3** in the visible region, with the main contributions to the 510 nm absorption shown on the right.

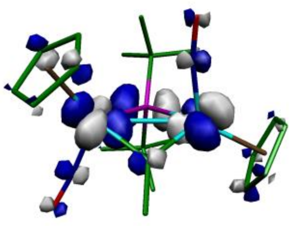

MO114 / –1.65 eV

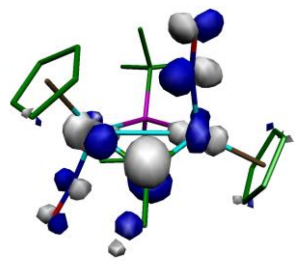

MO113(LUMO) / –2.43 eV

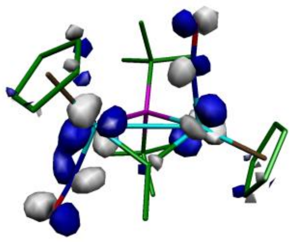

MO112(HOMO) / –4.51 eV

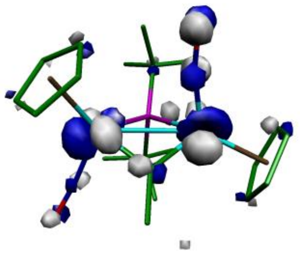

MO111 / –4.66 eV

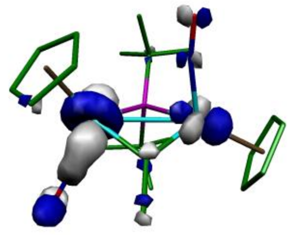

MO110 / –4.87 eV

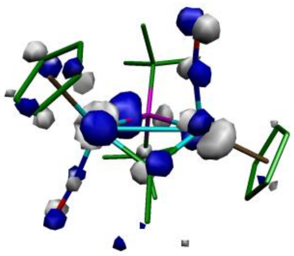

MO109 / –5.14 eV

Figure S20. Selected M06L-DFT-computed frontier molecular orbitals for compound **3**, with their energies (in eV) shown below.

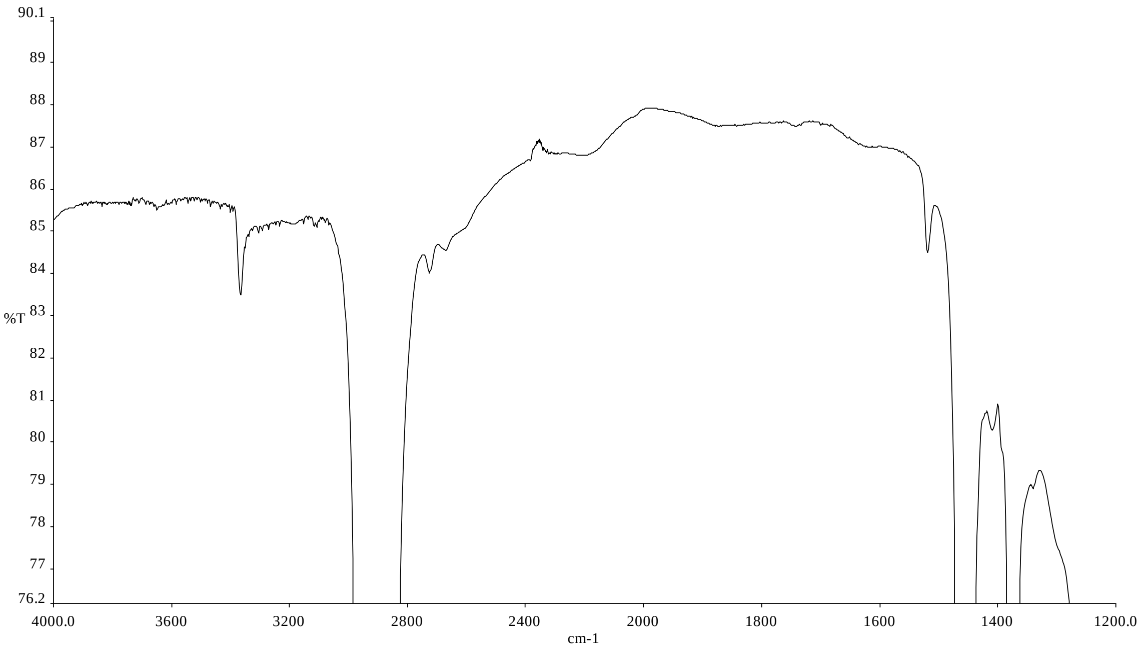

Figure S21. IR spectrum of compound **4-PF_6** in Nujol mull.

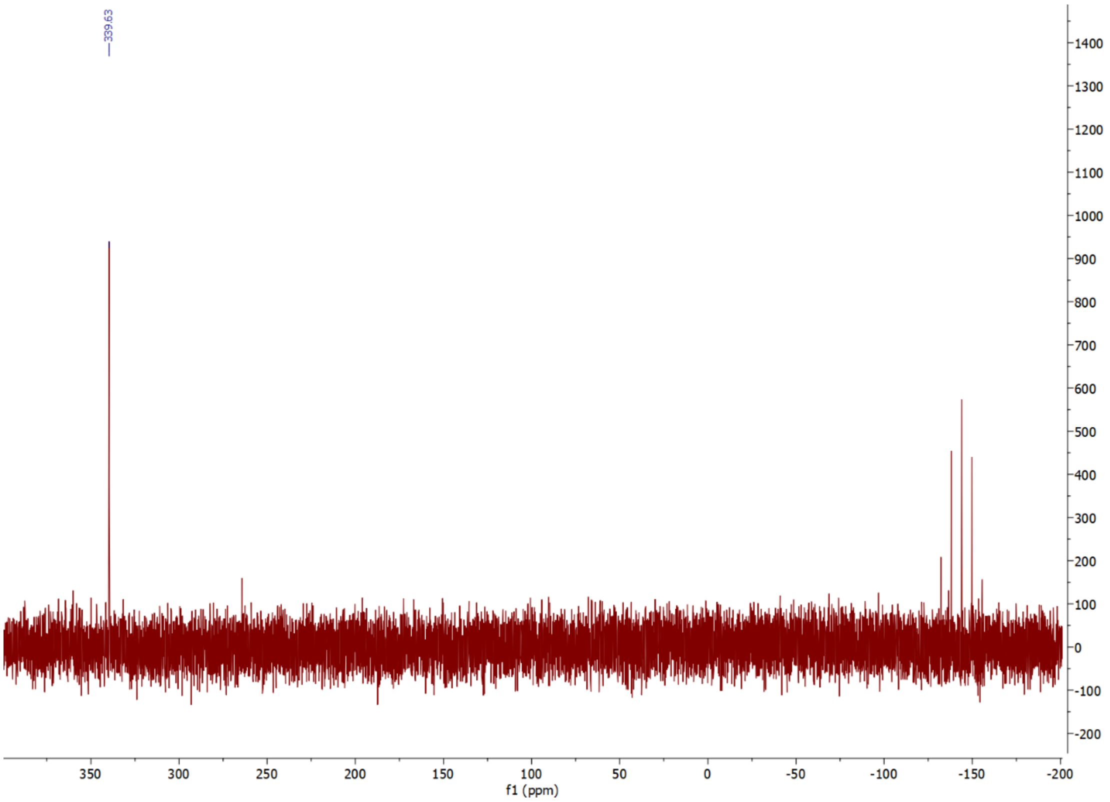

Figure S22. $^{31}P\{^1H\}$ NMR spectrum of compound **4-PF_6** (CD_2Cl_2).

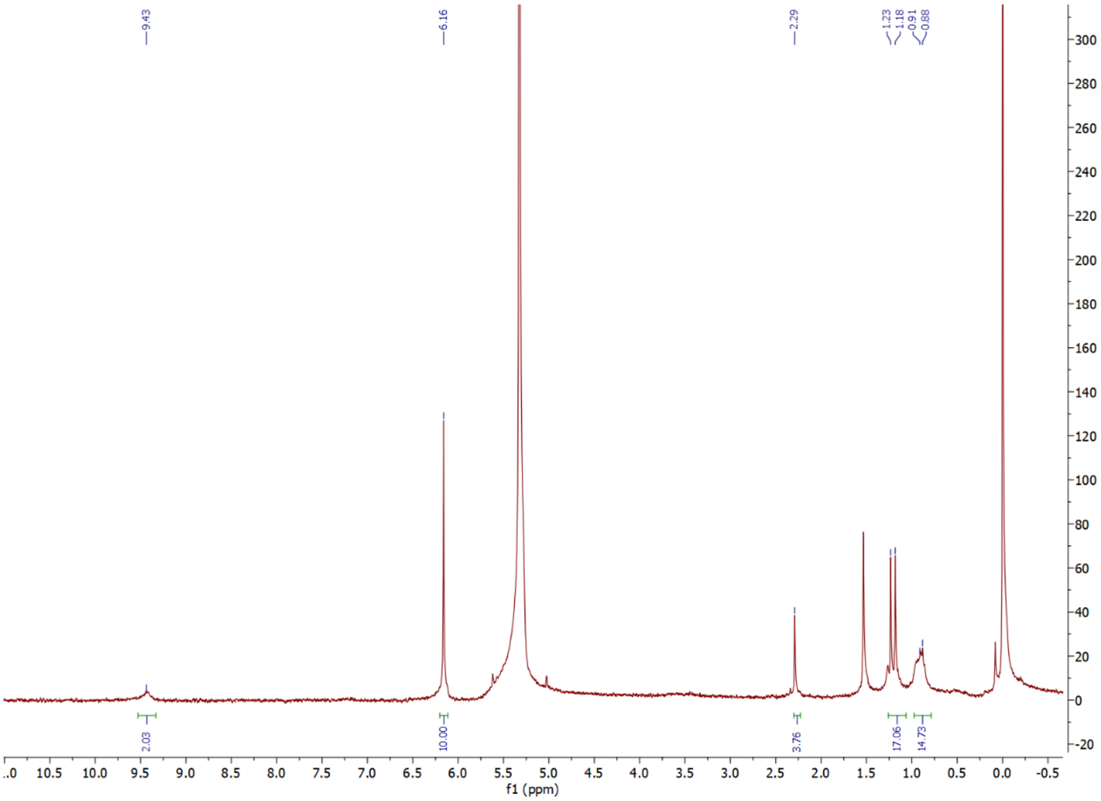

Figure S23. ^{1}H NMR spectrum of compound **4-PF$_6$** (CD_2Cl_2).

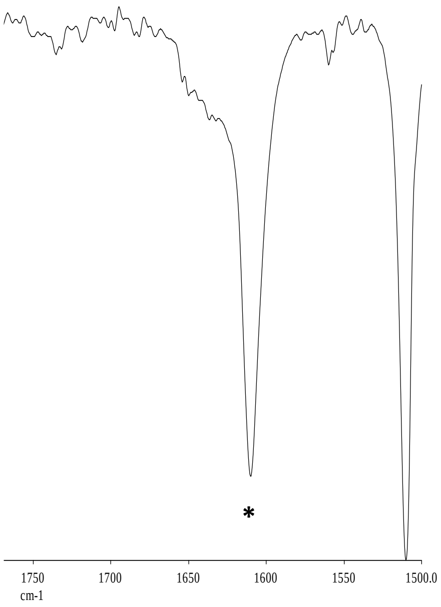

Figure S24. IR spectrum of compound **4-BAr4** in dichloromethane solution (the band marked with * corresponds to the BAr_4^- anion).

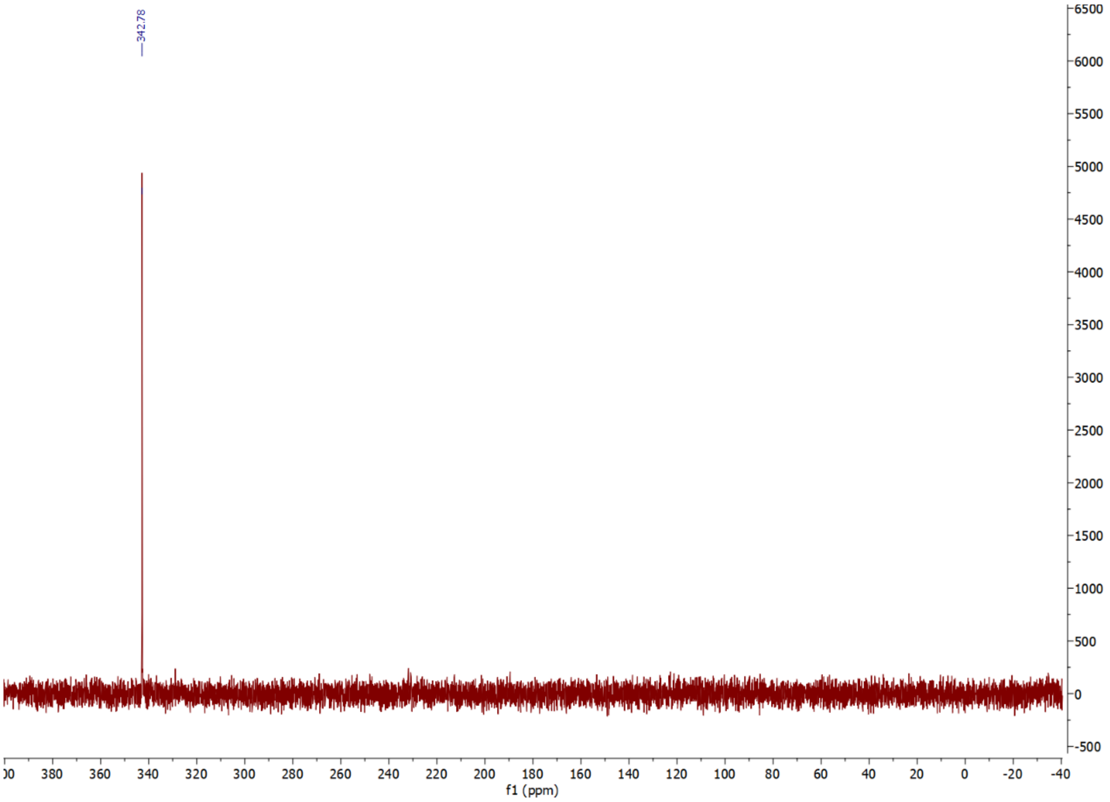

Figure S25. $^{31}P\{^1H\}$ NMR spectrum of compound **4-BAr4** (CD_2Cl_2).

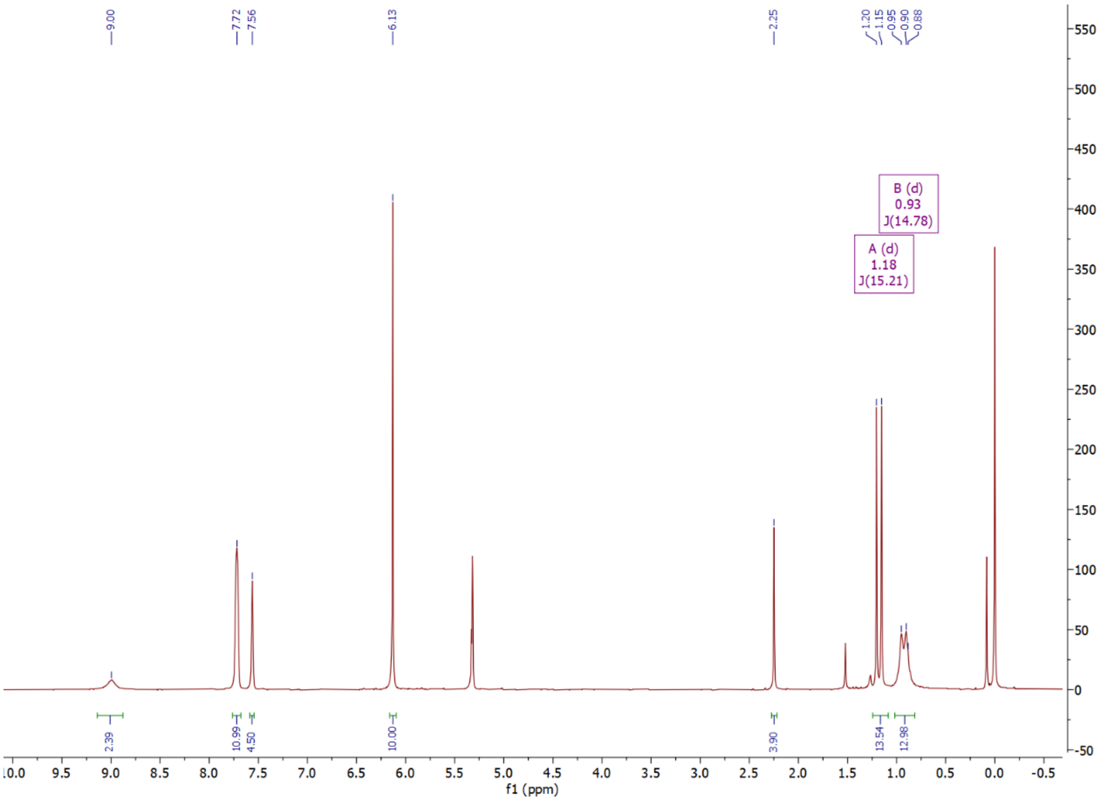

Figure S26. ^{1}H NMR spectrum of compound **4-BAr$_4$** (CD_2Cl_2).

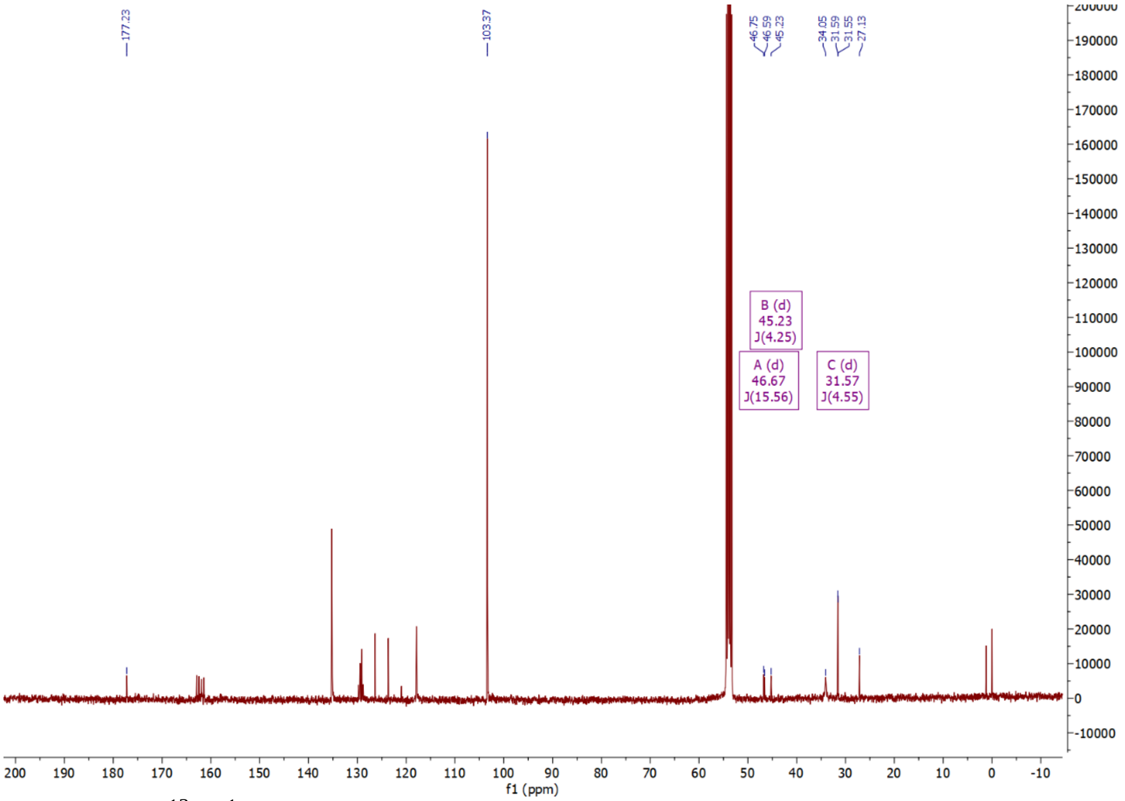

Figure S27. ^{13}C{^{1}H} NMR spectrum of compound **4-BAr$_4$** (CD_2Cl_2).

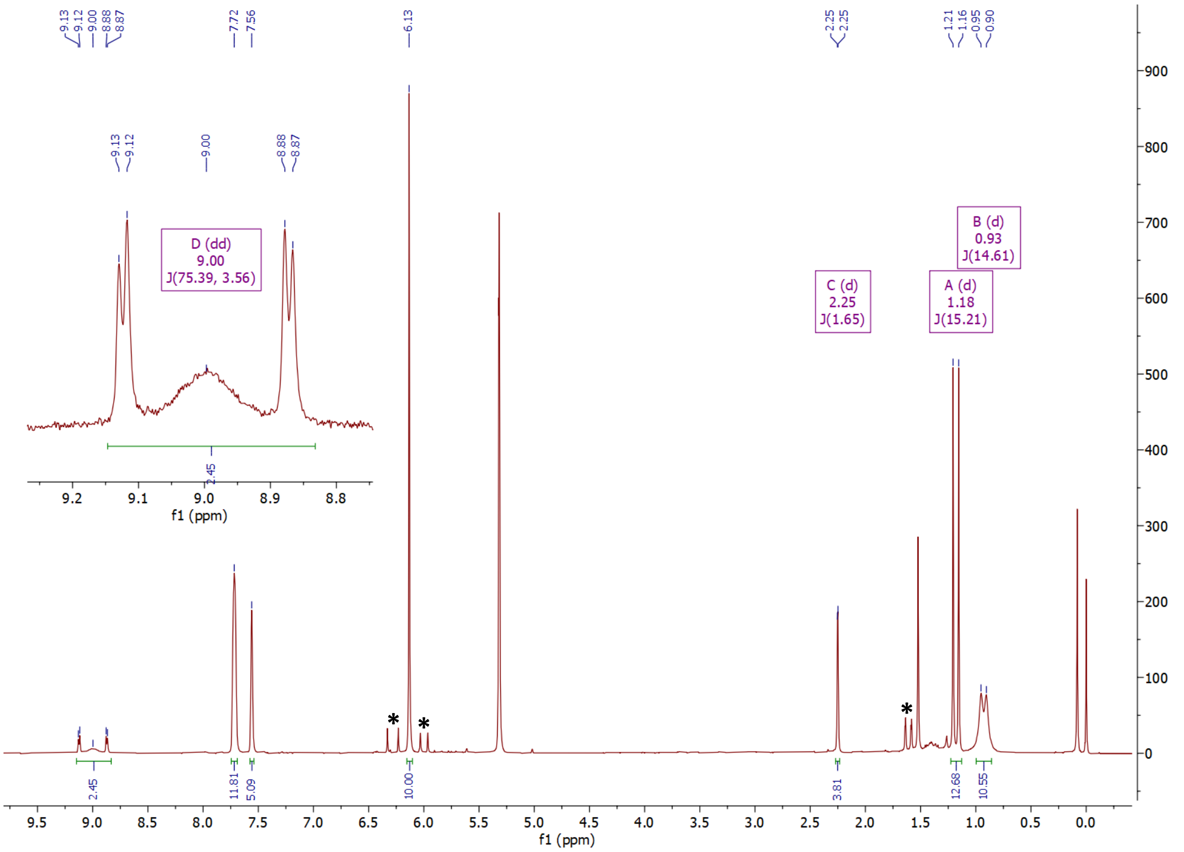

Figure S28. ^{1}H NMR spectrum of compound **4-BAr$_4$**, when prepared in 98% ^{15}N-enriched acetonitrile (CD_2Cl_2). The ^{15}N-bound H atom gives rise to a dd resonance at 9.00 ppm with $^{1}J_{H15N}$ = 75.4 Hz and $^{4}J_{HH}$ = 3.6 Hz, while the ^{14}N-bound H atom accounts for the quadrupolar-broadened resonance observed at the same chemical shift. The peaks marked with * correspond to minor impurities in the sample.
